# A Molecular Engineering Strategy to Fine‐Tune Phototoxicity of AIE Probes for Super‐Resolution Imaging of Mitochondrial Cristae Dynamics

**DOI:** 10.1002/advs.202524281

**Published:** 2026-07-06

**Authors:** Kongqi Chen, Mengjie Wang, Chuen Kam, Zhiming Wang, Qinrong Zhang, Sijie Chen

**Affiliations:** ^1^ School of Life Sciences The Chinese University of Hong Kong Hong Kong China; ^2^ AIE Institute Key Laboratory of Luminescence from Molecular Aggregates of Guangdong Province State Key Laboratory of Luminescent Materials and Devices South China University of Technology Guangzhou China; ^3^ Department of Biomedical Engineering City University of Hong Kong Hong Kong China; ^4^ AoE Centre for Plant Vacuole Biology and Biotechnology The Chinese University of Hong Kong Shatin Hong Kong China

**Keywords:** aggregation‐induced emission, fluorescent probes, mitochondrial ultrastructure, phototoxicity, super‐resolution imaging

## Abstract

Understanding mitochondrial morphology and function is critical for investigating mitochondrial diseases, yet fluorescent probes that label the inner mitochondrial membrane (IMM) and report dysfunction at high spatiotemporal resolution remain limited. Here, we rationally designed aggregation‐induced emission‐active probes with tunable alkyl chain lengths and carboxyl groups, allowing us to fine‑tune phototoxicity and achieve a controlled, mild level of reactive oxygen species (ROS) generation. Two probes, named OTS‐7C and OTS‐12C, enabled super‑resolution imaging of mitochondrial cristae using stimulated emission depletion microscopy (STED), while OTS‐12C further supported Hessian‐structured illumination microscopy (Hessian‐SIM) imaging in living cells. Importantly, the controllable ROS output of OTS‑12C supports prolonged time‐lapse imaging of mitochondrial stress responses, including swelling, cristae remodeling, and recovery. We further demonstrated that it could serve as a platform for evaluating antioxidant effectiveness, using Vitamin C and Astaxanthin as model antioxidants. Meanwhile, fluorescence lifetime imaging of OTS‐12C revealed that ROS‐induced oxidation of unsaturated lipids increased its fluorescence lifetime within the IMM, permitting real‐time monitoring of mitochondrial functional states. This work presents a simple yet effective strategy to fine‐tune the phototoxicity of AIE photosensitizers and provides OTS‐12C as a versatile fluorescent probe for high‐resolution visualization of mitochondrial ultrastructure, investigating mitochondrial stress management and evaluating drug antioxidant efficacy in living cells.

## Graphical abstract

Regulation of molecular packing, phototoxicity, and ROS output of AIE photosensitizers is achieved through carboxylation and alkyl‐chain‐length tuning. The probe designed via this strategy enables super‐resolution visualization of inner‐mitochondrial‐membrane cristae dynamics under controlled oxidative stress, and fluorescence‐lifetime imaging of mitochondrial functional states.

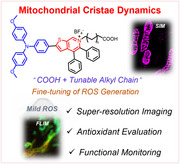

## 1 Introduction

Mitochondria are essential organelles serving as the primary site of ATP synthesis and acting as central hubs for cellular signaling in eukaryotic cells. They regulate numerous fundamental cellular processes, including apoptosis, autophagy, calcium homeostasis, stem cell differentiation, and immune response modulation [1, 2, 3, 4]. Structurally, mitochondria are characterized by a double‐membrane architecture composed of an outer mitochondrial membrane (OMM) and an inner mitochondrial membrane (IMM). The OMM acts as a selective diffusion barrier and a platform for signal transduction, whereas the IMM, comprising the inner boundary membrane and highly folded cristae, maximizes surface area to accommodate the molecular machinery required for oxidative respiration [5]. This intricate ultrastructure underpins the functional versatility of mitochondria [6]. Over decades, transmission electron microscopy (TEM) has been the gold standard for resolving the mitochondrial membrane ultrastructure owing to its exceptional spatial resolution [7, 8]. However, the requirement for specimen fixation and sectioning in TEM precludes real‐time observation of dynamic processes in living cells. Recent advancements in live‐cell compatible super‐resolution (SR) microscopy, such as stimulated emission depletion (STED) microscopy and structured illumination microscopy (SIM), have therefore opened new opportunities to study mitochondrial dynamics. STED microscopy routinely achieves lateral resolution of approximately 40–50 nm, whereas SIM typically has a resolving power of 100–120 nm [9, 10]. These approaches enable direct visualization and tracking of cristae remodeling in living cells.

SR imaging depends heavily on the optimal photophysical properties of fluorophores to achieve high resolution, and the development of bright and photostable fluorescent probes optimized for SR applications remains an active area of research. However, imaging of the mitochondrial membrane is particularly challenging because fluorescence excitation can generate reactive oxygen species (ROS) [11]. Strategies that increase fluorescence yield by prolonging or increasing excitation light can therefore also induce mitochondrial stress by enhanced ROS production, which potentially activates apoptotic pathways [12]. To tune the photophysical properties of fluorescent probes, such as the emission wavelength, brightness, and excitation efficiency, a common chemical approach is to incorporate donor (D) and acceptor (A) moieties into the probe to form a typical donor–π–acceptor (D–π–A) structure. This structural modification effectively reduces the singlet–triplet energy gap (ΔE_S‐T_) and enhances brightness, but can also increase ROS generation [13, 14]. Recent efforts to mitigate phototoxicity associated with fluorescent dyes have included triplet‐state engineering of cyanine‐based mitochondrial probes by conjugating with cyclooctatetraene (COT) [15, 16]. While this strategy yields improved performance in mitochondrial STED imaging (e.g., PK Mito Red/Orange), the complex synthesis procedure is time‐consuming. Alternatively, the use of 9,9‐dimethyl‐9,10‐dihydroacridine (DMA) as an electron‐donating auxochrome enhances photostability and reduces phototoxicity, but it has not yet enabled clear visualization of mitochondrial cristae [17].

Notably, although previous studies have achieved excellent STED resolution of cristae by using optimized probes and imaging conditions, these efforts have largely focused on minimizing phototoxicity to preserve sample integrity. In contrast, the dynamics of mitochondrial cristae in response to ROS remain poorly understood, which limits insights into related pathological conditions. A key challenge is therefore to balance the intensity of ROS stimulation with imaging performance to enable effective observation of cristae dynamics, which is difficult to achieve with exogenous ROS alone and separately optimized imaging. Rather than seeking completely inert probes, an alternative strategy is to develop functional probes that generate controlled, mild ROS as a built‐in stimulus to study mitochondrial structural responses under oxidative stress.

In this study, we present a molecular design strategy that modulates phototoxicity by incorporating carboxyl groups (COOH) and by tuning the aggregation‐induced intersystem crossing (AI‐ISC) via chain‐length‐dependent molecular packing of aggregation‐induced emission (AIE) photosensitizers. This modification effectively suppresses triplet state formation and modulates the aggregation behavior, which significantly reduces ROS generation, allowing us to fine‐tune the phototoxicity of the molecules. Using this approach, we developed three AIE‐active probes, OTS‐4C, OTS‐7C, and OTS‐12C. OTS‐7C and OTS‐12C target mitochondria and can be used for SR imaging of mitochondrial cristae beyond the diffraction limit by both STED and SIM microscopy. Beyond imaging, we demonstrate that the controlled ROS generation of OTS‐12C can serve as a built‐in stimulus for antioxidant efficacy evaluation. Using Vitamin C and Astaxanthin as model antioxidants, we show that OTS‐12C enables visualizing the protective effects of antioxidants on mitochondria under mild oxidative stress, as evidenced by preserved cristae density and reduced mitochondrial width changes. This establishes OTS‐12C as a dual‐function probe for both super‐resolution visualization and nanoscale assessment of mitochondrial stress responses. Application of OTS‐12C enables visualization of mitochondrial membrane remodeling in response to external stimuli, thereby enhancing our understanding of mitochondrial dynamics. Additionally, the incorporation of fluorescence lifetime imaging microscopy (FLIM) provides real‐time readout of mitochondrial functional states. Together, these results underscore the significant potential of OTS‐12C for advanced live‐cell mitochondrial analysis.

## 2 Results and Discussion

### 2.1 Molecular Design to Regulate Phototoxicity

**FIGURE 1:**
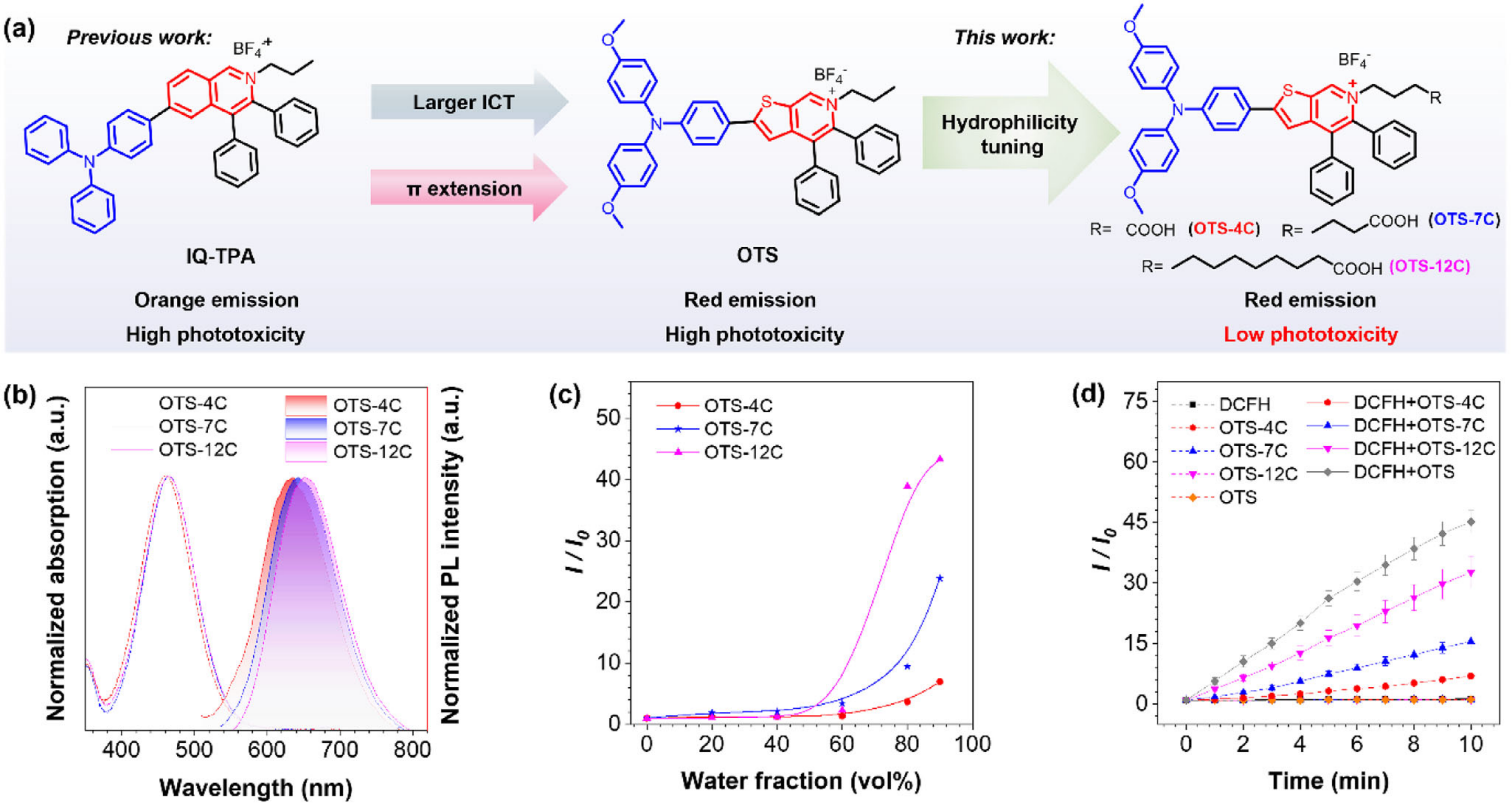
(a) Illustration of the design strategy of mitochondrial dyes based on previously reported IQ‐TPA. (b) Absorption spectra and fluorescence emission spectra of OTS‐4C, OTS‐7C, and OTS‐12C. (c) *I/I_0_* plots of OTS‐4C, OTS‐7C, and OTS‐12C in a mixture of DMSO/H_2_O, where *I* is the intensity with different H_2_O volume fractions (*f*_w_), and *I*_0_ is the initial intensity in DMSO. (d) Plots of the relative PL intensity of DCFH (for general ROS detection) in the presence of 10 µM OTS derivatives in PBS with 1 vol.% DMSO vs. irradiation time (white light, 30 mW•cm^−2^).

The D–π–A probe IQ‐TPA, comprising a cationic isoquinolinium core and an electron‐donating triphenylamine moiety, was previously reported to exhibit a large Stokes shift, high photostability, and mitochondrial targetability [18]. Despite having these favorable properties, IQ‐TPA exhibits substantial phototoxicity that limits its applications in long‐term and SR imaging of mitochondria. To address these limitations, we employed a systematic “step‐by‐step” molecular optimization strategy, as illustrated in Figure 1a. First, we introduced a methoxy group as an electron‐donating moiety into the triphenylamine structure to enhance intramolecular charge transfer (ICT) and further red‐shift both absorption and emission. Furthermore, the fused phenyl ring of the isoquinoline scaffold was replaced by a lipophilic π‐conjugated thiophene unit to extend the π‐conjugated backbone.

Because AI‐ISC can increase ROS production [19, 20], we next tuned the aggregation property of the dye by incorporating a hydrophilic COOH group and alkyl chains of varying lengths into the molecular framework. These IQ‐TPA derivatives were synthesized by a straightforward one‐pot multicomponent reaction catalyzed by a rhodium complex, as outlined in Scheme S1. All synthesized compounds were characterized using ^1^H and ^13^C nuclear magnetic resonance spectroscopy, as well as high‐resolution mass spectrometry (ESI) spectral analysis (Figures S1–S6).

### 2.2 Photophysical Properties and ROS Generation Efficiency

The absorption spectra of OTS‐4C, OTS‐7C, and OTS‐12C are shown in Figure 1b. In pure dimethyl sulfoxide (DMSO) solution, these derivatives displayed comparable absorption maxima (462, 465, and 465 nm, respectively), whereas their emission maxima in 99 vol.% *dd*H_2_O were progressively redshifted (634, 642, and 652 nm, respectively). The solvent‐dependent redshift in their emission spectra is attributed to the altered aggregation behavior driven by the introduced alkyl chains and the COOH group. To further investigate their AIE characteristics, DMSO was used as a good solvent and water as a poor solvent for these organic molecules. As depicted in Figure 1c, fluorescence intensity increased with an increasing water fraction (*f*_w_). In pure DMSO, these probes were weakly emissive owing to their strong ICT feature and excellent solubility in DMSO. Notably, at *f*_w_ = 90%, OTS‐12C exhibited a 42‐fold fluorescence enhancement relative to pure DMSO, while OTS‐7C and OTS‐4C displayed approximately 25 and 8‐fold increases, respectively (Figure S7). These results confirm the typical AIE behavior of these three compounds.

Because ROS generation is a key determinant of phototoxicity, we quantified total ROS production using 2’,7’‐dichlorofluorescein (DCFH) as an indicator. Solutions containing 10 µM OTS, OTS‐4C, OTS‐7C, OTS‐12C, the control parent compound OTS, and the control photosensitizer Rose Bengal (RB) were illuminated with white light at a power density of 30 mW•cm^−2^, and the fluorescence of oxidized DCF was monitored at 525 nm. As depicted in Figure 1d and Figure S8a, OTS exhibited the largest increase in DCF fluorescence intensity after 10 min of illumination, followed by OTS‐12C, RB, OTS‐7C, and OTS‐4C. These results indicate that OTS has the highest ROS generation rate and align well with our design hypothesis that the COOH group mitigates ROS generation. To further dissect ROS types, 9,10‐anthracenediyl bis(methylene) dimalonic acid (ABDA) and hydroxyphenyl fluorescein (HPF) were employed to detect singlet oxygen (^1^O_2_) and hydroxyl radicals (•OH), respectively. After 5 min of light irradiation, ABDA absorbance decreased by 18% (OTS), 16% (OTS‐4C), 16% (OTS‐7C), 20% (OTS‐12C), and 28% (RB) (Figure S8b). The rank order differs from the DCFH results because RB is predominantly a Type‐II photosensitizer, whereas our probes generate mixed ROS species. Importantly, this assay confirms that all carboxyl‐containing probes produce less singlet oxygen than RB, directly supporting our design strategy of mitigating phototoxic ROS. Additionally, the photoluminescence (PL) intensities of HPF were measured to assess Type‐I ROS generation through electron transfer from excited molecules to oxygen [21]. The fluorescence intensity markedly showed the greatest increase within 8 min for OTS under light illumination, followed by OTS‐12C, OTS‐7C, and OTS‐4C (Figure S8c). This trend resembles the total ROS generation profile, indicating that the COOH moiety selectively suppresses Type‐I ROS formation. These data suggest that the COOH moiety hampers initial radical species formation.

### 2.3 Mechanistic Study of ROS Generation Efficiency Modulation via Molecular Engineering

**FIGURE 2:**
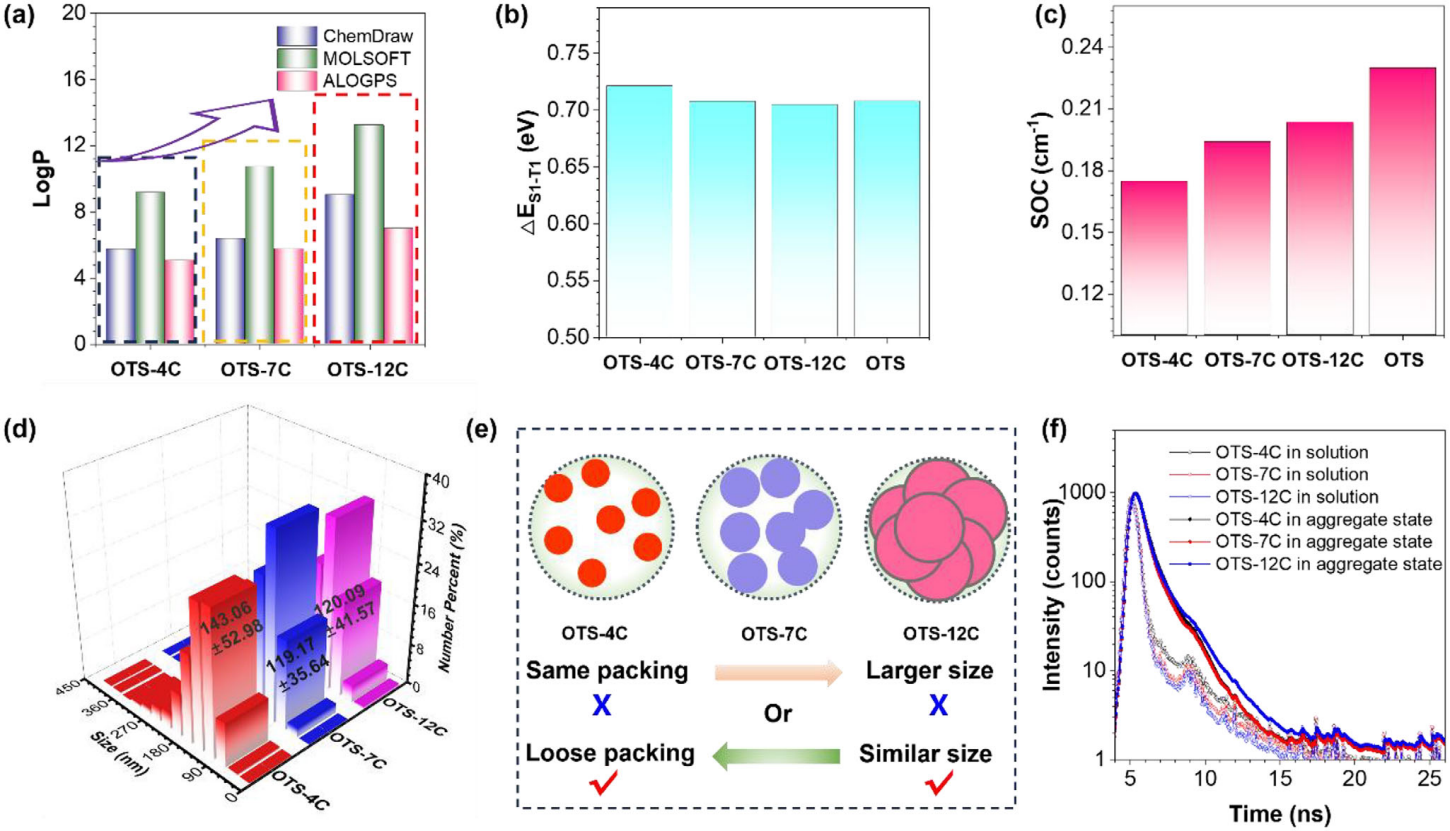
(a) Calculated values of Log P based on different software. (b) ΔE_S‐T_ and (c) SOC values of OTS, OTS‐4C, OTS‐7C and OTS‐12C based on density functional theory calculations. (d) Hydrodynamic diameters and distribution of OTS‐4C, OTS‐7C and OTS‐12C in 99 vol.% *dd*H_2_O. (e) Schematic illustration of the relationship between packing modes and nanoparticle sizes. (f) Time‐resolved fluorescence decay of OTS‐4C, OTS‐7C and OTS‐12C in solution and aggregated state.

Computational analysis of the coefficient (Log P) values using multiple methods (Figure 2a) indicates that OTS derivatives bearing longer alkyl chains and a COOH group are increasingly hydrophobic. This trend reflects the influence of alkyl chain length on the balance between hydrophilic and lipophilic properties. We therefore performed theoretical calculations to evaluate the Δ*E*_S‐T_ and the spin−orbit coupling (SOC) value between the singlet (S_1_) and nearby triplet (T_1_ or T_2_) states due to their important roles in ROS generation. Incorporation of the COOH group had a negligible effect on ΔE_S‐T,_ which indicates that the energy gap remains largely unchanged. However, the COOH group significantly reduced the SOC value, with a more pronounced decrease observed in derivatives with shorter alkyl chains (Figure 2b,c). These results imply that suppression of SOC primarily contributes to the reduced ROS production for derivatives with shorter alkyl chains.

We next measured the hydrodynamic sizes of OTS‐4C, OTS‐7C, and OTS‐12C in aqueous solution using dynamic light scattering. The more hydrophilic OTS‐4C had a larger size (143.06 ± 52.98 nm) compared to OTS‐7C (119.17 ± 35.64 nm) and OTS‐12C (120.09 ± 41.57 nm) (Figure 2d), which is in accord with the results of aggregation morphology analyzed by a scanning electron microscope (SEM) (Figure S9). On the basis of these structural and size data, we hypothesize that differences in aggregate structure underlie the varying ROS generation efficiencies among these derivatives (Figure 2e). Fluorescence lifetime, defined as the average time a fluorophore remains in its excited state, is sensitive to the local environment and serves as a metric for studying molecular aggregation [22]. Accordingly, the fluorescence lifetimes of OTS‐4C, OTS‐7C, and OTS‐12C were measured in both dispersed and aggregated states. As shown in Figure 2f, the fluorescence lifetime of OTS derivatives in the dispersed state decreased with longer alkyl chains bearing the COOH group (0.41, 0.27, and 0.22 ns for OTS‐4C, OTS‐7C, and OTS‐12C, respectively). Conversely, an opposite trend was observed in the aggregated state (0.76, 0.72, and 0.85 ns for OTS‐4C, OTS‐7C, and OTS‐12C, respectively). Taken together with the AIE properties of these probes, these data indicate that tighter molecular packing in the aggregated state correlates with longer fluorescence lifetimes. Consequently, shorter alkyl chains with the COOH group favor looser packing and reduced ROS generation, which is consistent with the AI‐ISC principles. This mechanistic insight suggests that molecular design strategies promoting looser aggregation can mitigate phototoxicity in SR imaging. Together, these two mechanisms operate synergistically: the carboxyl group provides baseline SOC suppression across all probes, while chain‐length‐dependent packing fine‐tunes the AI‐ISC contribution. The result is that OTS‐4C benefits from both strong SOC suppression and minimal AI‐ISC, yielding the lowest ROS, whereas OTS‐12C partially recovers ROS due to tighter packing enhancing AI‐ISC, while still maintaining reduced SOC relative to unmodified OTS.

### 2.4 Cellular Phototoxicity and Photostability

**FIGURE 3:**
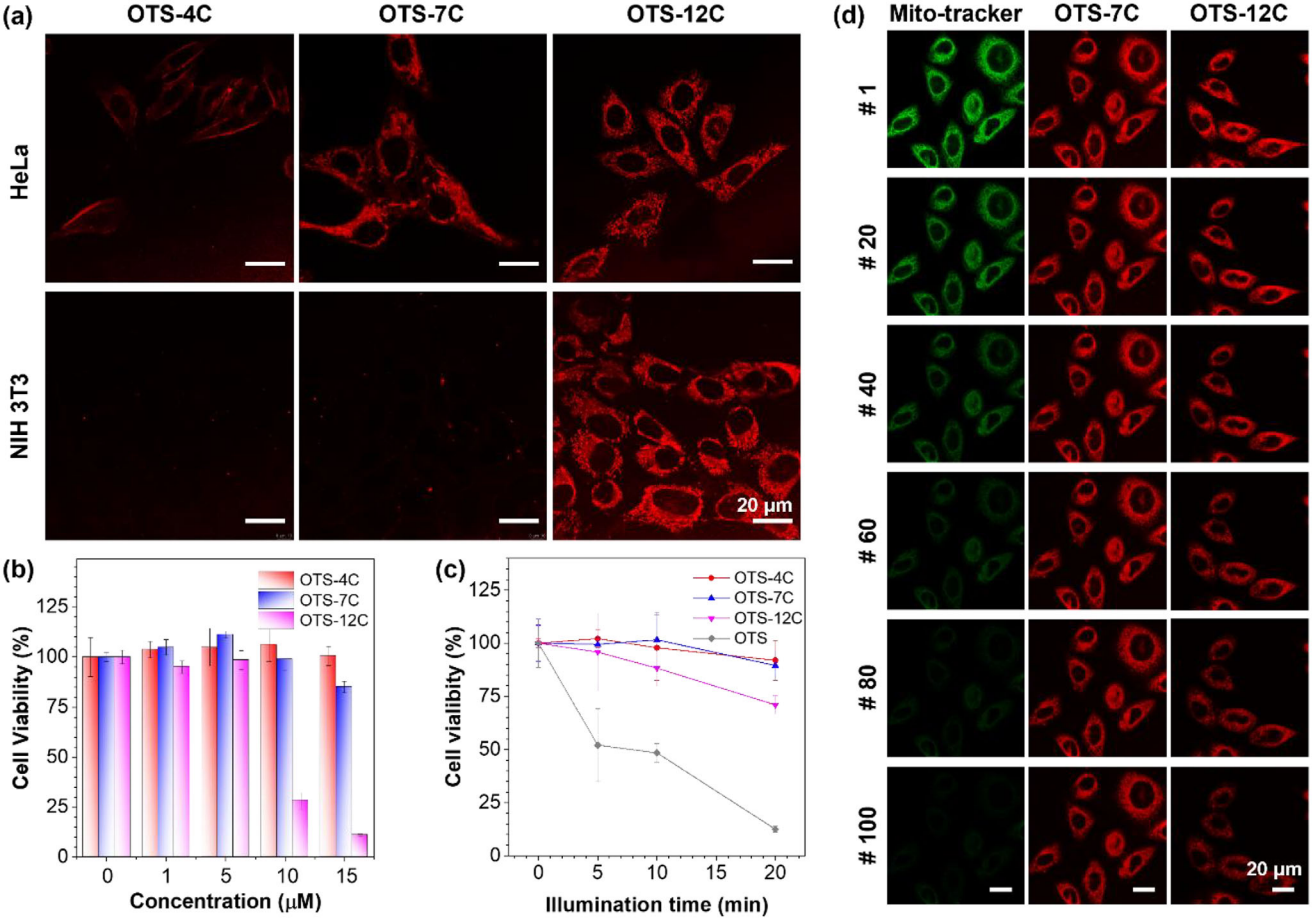
(a) Confocal fluorescence images of living HeLa and NIH/3T3 cells incubated with 5 µM OTS‐4C, OTS‐7C and OTS‐12C for 30 min. (b) Cell viability of HeLa cells incubated with varied concentrations of OTS‐4C, OTS‐7C and OTS‐12C for 24 h. (c) Phototoxicity of OTS, OTS‐4C, OTS‐7C and OTS‐12C in HeLa cells measured by cell survival rates after white light illumination for different periods of time. (d) Confocal fluorescence images in indicated iterations of HeLa cells labeled with Mito‐tracker Green, OTS‐7C or OTS‐12C over 10 min by a confocal laser scanning microscope. Selected frames (#1, #20, #40, #60, #80, #100) from a 100‐frame time‐lapse sequence obtained by continuous epifluorescence scanning.

To evaluate the biocompatibility of OTS‐4C, OTS‐7C, and OTS‐12C, we investigated their effects on cell viability using the cell counting kit‐8 (CCK‐8) assays. In the absence of light illumination, HeLa cells treated with up to 5 µM of each OTS derivative for 24 h exhibited no significant decrease in the dehydrogenase activity (Figure 3b), indicating minimal cytotoxicity and high biocompatibility in live cells. Additionally, a pulse exposure experiment (1 h treatment followed by 24 h incubation) confirmed that even at 10 µM of OTS‐12C, cell viability remained above 90% (Figure S10), demonstrating that the probe has no significant toxicity within the relevant short‐term imaging window. The low phototoxicity of these OTS derivatives prompted us to further evaluate their cellular uptake and subcellular distribution in HeLa (a cancer cell line) and NIH/3T3 cells (a normal cell line). As shown in Figure 3a, HeLa cells incubated with 5 µM OTS‐4C, OTS‐7C, or OTS‐12C for 30 min displayed clear intracellular fluorescence signals. Conversely, NIH/3T3 cells showed undetectable intracellular signals under identical imaging settings, with some bright extracellular puncta for OTS‐4C and OTS‐7C. Notably, OTS‐12C exhibited comparable fluorescence signals in both HeLa and NIH/3T3 cells, suggesting non‐selective uptake. These results indicate that OTS‐4C and OTS‐7C are preferentially internalized by cancer cells, possibly due to differences in hydrophobicity, aggregation behavior discussed earlier, and surface charge characterized by zeta potential (Figure S11). To further elucidate their subcellular localization, colocalization experiments were conducted (Figure S12). OTS‐4C predominantly labeled the plasma membrane while OTS‐7C and OTS‐12C specifically targeted mitochondria, highlighting their potential as mitochondrial probes for SR imaging. MitoTracker Green (MTG) was employed as a commercial reference to confirm general mitochondrial localization. Subsequent super‐resolution imaging and analysis were then performed to specifically demonstrate the probe's superior capability to resolve the IMM and cristae architecture. In addition, fluorescence signals from cells co‐stained with OTS‐4C, OTS‐7C, OTS‐12C, and markers for ER (ER‐Tracker) and lysosomes (LysoTracker Deep Red) show minimal correlation (Figures S13 and S14).

To evaluate the phototoxicity of OTS derivatives at the cellular level, we utilized the CCK‐8 assay to measure HeLa cell viability following treatment with 5 µM OTS, OTS‐4C, OTS‐7C, or OTS‐12C and exposure to varying durations of illumination. Figure 3c illustrates that HeLa cells treated with OTS exhibited substantial cell death after approximately 5 min of illumination, with nearly 90% cell death after 20 min. In contrast, cells treated with OTS‐4C, OTS‐7C, and OTS‐12C retained a high viability after 5 min of illumination, with cell death rates of only 10%, 10%, and 20%, respectively, after 20 min of prolonged illumination. These results demonstrate that the incorporation of the COOH group markedly mitigates cellular photodynamic damage induced by extended light exposure. Because of its high phototoxicity, OTS was excluded from further analysis in this study.

Furthermore, the photostability of OTS‐7C and OTS‐12C was evaluated in comparison to the commercial MitoTracker Green under identical imaging conditions. As depicted in Figure 3d, MitoTracker Green exhibited over 95% fluorescence loss after 100 iterations due to significant photobleaching. In contrast, OTS‐7C and OTS‐12C demonstrate superior photostability, retaining 90% and 70% of their initial fluorescence signals, respectively (Figure S15). Importantly, neither OTS‐7C nor OTS‐12C altered mitochondrial morphology, including overall shape or branch length, during the imaging process. These findings highlight the exceptional photostability and low phototoxicity of OTS‐7C and OTS‐12C in a biological context, rendering them highly suitable for extended live‐cell STED imaging.

### 2.5 Super‐Resolution Imaging of Mitochondria

**FIGURE 4:**
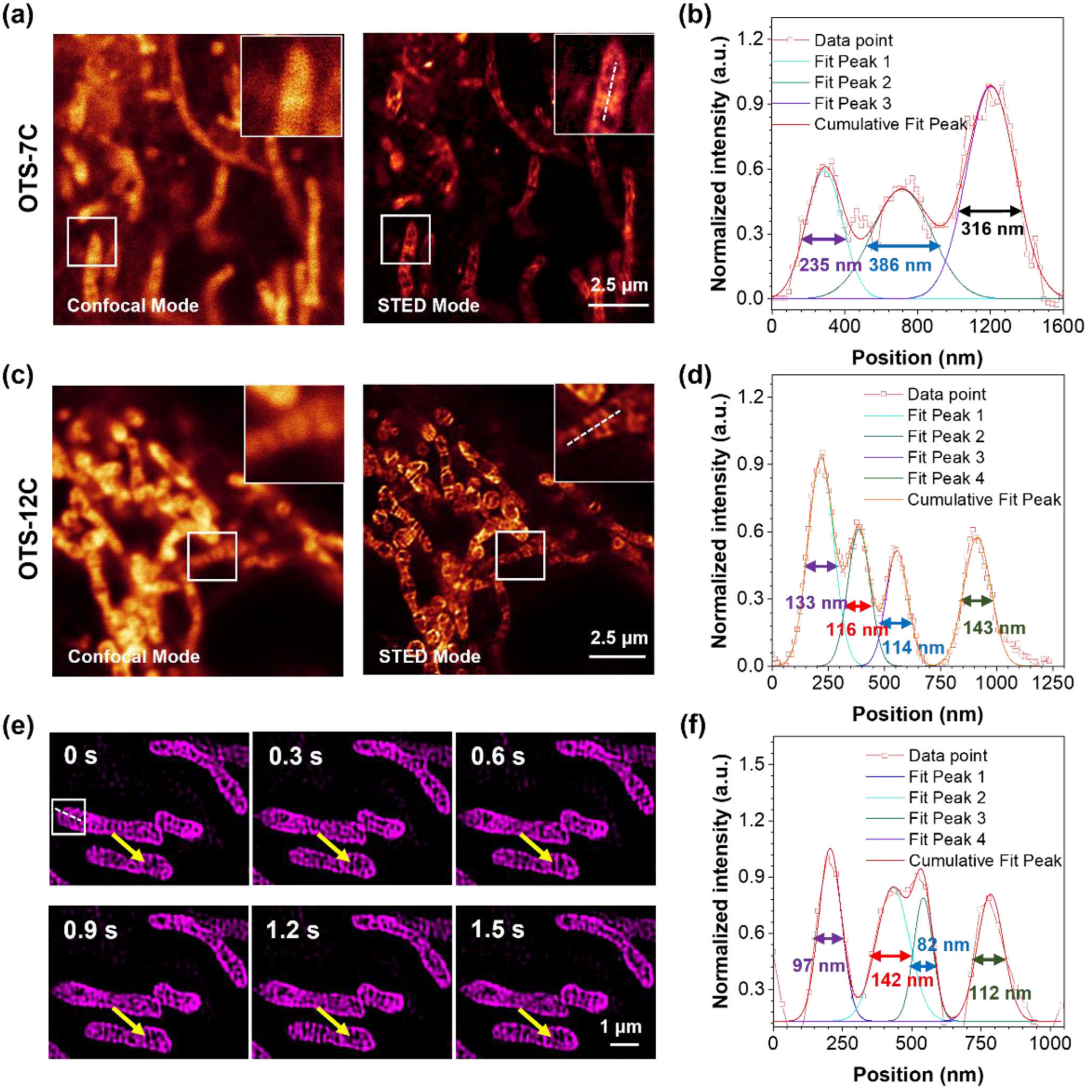
Fluorescence images acquired by confocal microscopy and STED microscopy and their corresponding intensity profiles along the lines across cristae in HeLa cells stained with OTS‐7C (a,b) or OTS‐12C (c,d). The insets are their corresponding enlarged views. (e) HeLa cells stained with OTS‐12C were imaged using Hessian‐SIM in time‐lapse mode, and (f) the corresponding intensity profiles along the lines across cristae at the 0 s time point.

The remarkable photostability and low phototoxicity of OTS‐7C and OTS‐12C make them potential probes for SR imaging of mitochondria by STED microscopy. The absorption and emission spectra of OTS‐7C and OTS‐12C (Figure 1b) are compatible with 488 nm excitation and 775 nm STED depletion, and HeLa cells stained with 5 µM OTS‐7C or OTS‐12C produced high‐quality images on a commercial STED system. Notably, both probes revealed a distinct ladder‐like pattern of mitochondrial cristae that was not resolved by confocal imaging (Figure 4a,c), indicating preferential localization of these probes to the IMM in living cells. Lateral resolution was quantified from full‐width at half maximum (FWHM) measurements of fluorescence intensity profiles across individual cristae in STED images. As shown in Figure 4b, the FWHMs were 235, 386, and 316 nm for OTS‐7C and 133, 116, 114, and 143 nm for OTS‐12C (Figure 4d). These results demonstrate that OTS‐12C achieves adequate resolution for cristae visualization, possibly correlating with its improved mitochondrial membrane selectivity and higher brightness supported by photophysical characterization.

Given the performance of OTS‐12C in STED imaging of mitochondria, we further evaluated its compatibility with SIM to demonstrate its versatility across different SR imaging modalities. SIM increases the spatial resolution of wide‐field fluorescence microscopy while enabling high frame‐rate acquisition with relatively low photodamage, making it particularly well‐suited for live‐cell studies [23, 24]. By using SIM, we successfully visualized clear mitochondrial cristae in HeLa cells stained with OTS‐12C (Figure S16). High‐speed time‐lapse imaging readily resolves dynamic mitochondrial cristae morphology, as indicated by the yellow arrows in Figure 4e. The FWHMs were 97, 142, 82, and 112 nm for OTS‐12C under SIM (Figure 4f). These time‐lapse images underscore the ability of OTS‐12C to support imaging of mitochondrial ultrastructure in living cells at both high spatial and temporal resolution.

### 2.6 Time‐Lapse SIM Imaging of Mitochondria Under Stress

**FIGURE 5:**
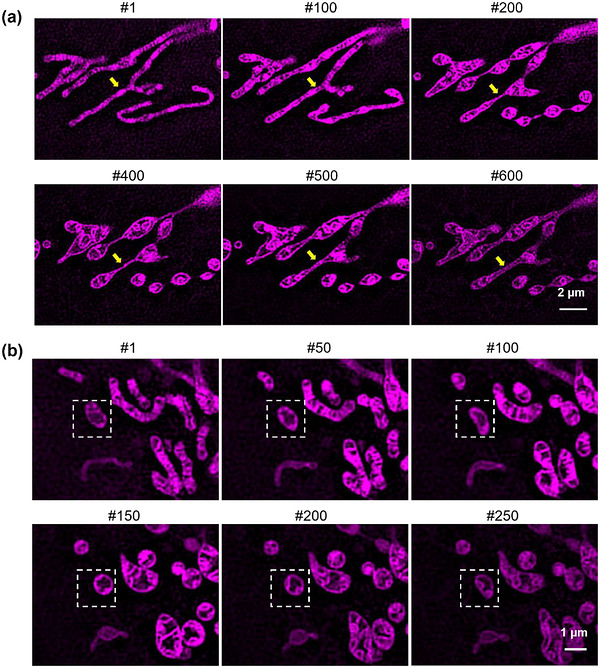
(a and b) Time‐lapse SR imaging of mitochondria in U2OS cells using Hessian‐SIM under different laser intensities. Images were acquired at 9.26 fps, the 488 nm laser with an intensity of 2 mW (a) and 6.4 mW (b), respectively. Selected frames (#1, #100, #200, #400, #500, #600) from a 600‐frame continuous time‐lapse sequence under constant epifluorescence illumination.

Consistent with our molecular design strategy of fine‐tuning phototoxicity, OTS‐12C behaves as a weak photosensitizer: light irradiation under controlled power and duration enables controlled ROS generation. Therefore, we can induce mild photodamage to mitochondria. This allows the study of mitochondrial morphology dynamics under mild oxidative conditions. To investigate the dynamics of mitochondrial cristae under ROS influence during light exposure, U2OS cells labeled with OTS‐12C were imaged by time‐lapse SIM at a frame rate of 9.26 frames per second (fps). Over more than 200 consecutive frames, we observed notable changes in mitochondrial morphology without loss of fluorescence intensity (Figure 5a and Figure S17). Mitochondria initially appeared as tubular structures characterized by reduced matrix density and cristae. These structures subsequently transformed into an elongated and dumbbell‐shaped structure connected by an intact OMM. This midzone division process indicates healthy mitochondrial proliferation with minimal photodamage [25]. Interestingly, extended observation demonstrated that these dumbbell‐shaped mitochondria transformed back to tubular morphology instead of membrane fission. These results showed that mitochondria can rapidly and reversibly remodel under mild ROS‐induced stress. To the best of our knowledge, while electron microscopy has provided ultrastructural snapshots of mitochondrial recovery [26], this is the first real‐time visualization of this entire dynamic process—from stress‐induced morphological remodeling to the regeneration of cristae—within living cells.

Recent studies have identified two mitochondrial subpopulations with distinct functionality that coexist within cells: one enriched in cristae for oxidative phosphorylation and the other lacking cristae for reductive biosynthesis [27]. In Figure 5b, the cristae‐deficient mitochondria indicated in the white dashed boxes are clearly distinguishable from the neighboring cristae‐containing mitochondria. The structural dynamics of these subpopulations in live cells, particularly under mitochondrial stress, remain incompletely characterized. To probe stress‐induced remodeling, high‐power laser excitation was applied during SIM imaging to induce photo‐oxidative stress. Over 150 frames, both cristae‐containing and cristae‐deficient mitochondria exhibited swelling, which indicates stress‐related changes in mitochondrial membrane integrity (Figure 5b). Notably, extended imaging revealed *de novo* formation of cristae within the cristae‐deficient subpopulation. This observation indicates that cristae‐deficient mitochondria are capable of acquiring foldings in the IMM, a morphology associated with ATP production, in a dynamic transition under photo‐oxidative stress. Together, these findings reveal functional plasticity among mitochondrial subpopulations and highlight their adaptive mechanisms in response to mitochondrial stress.

### 2.7 Visualizing Mitochondrial Protective Effects of Antioxidants

**FIGURE 6:**
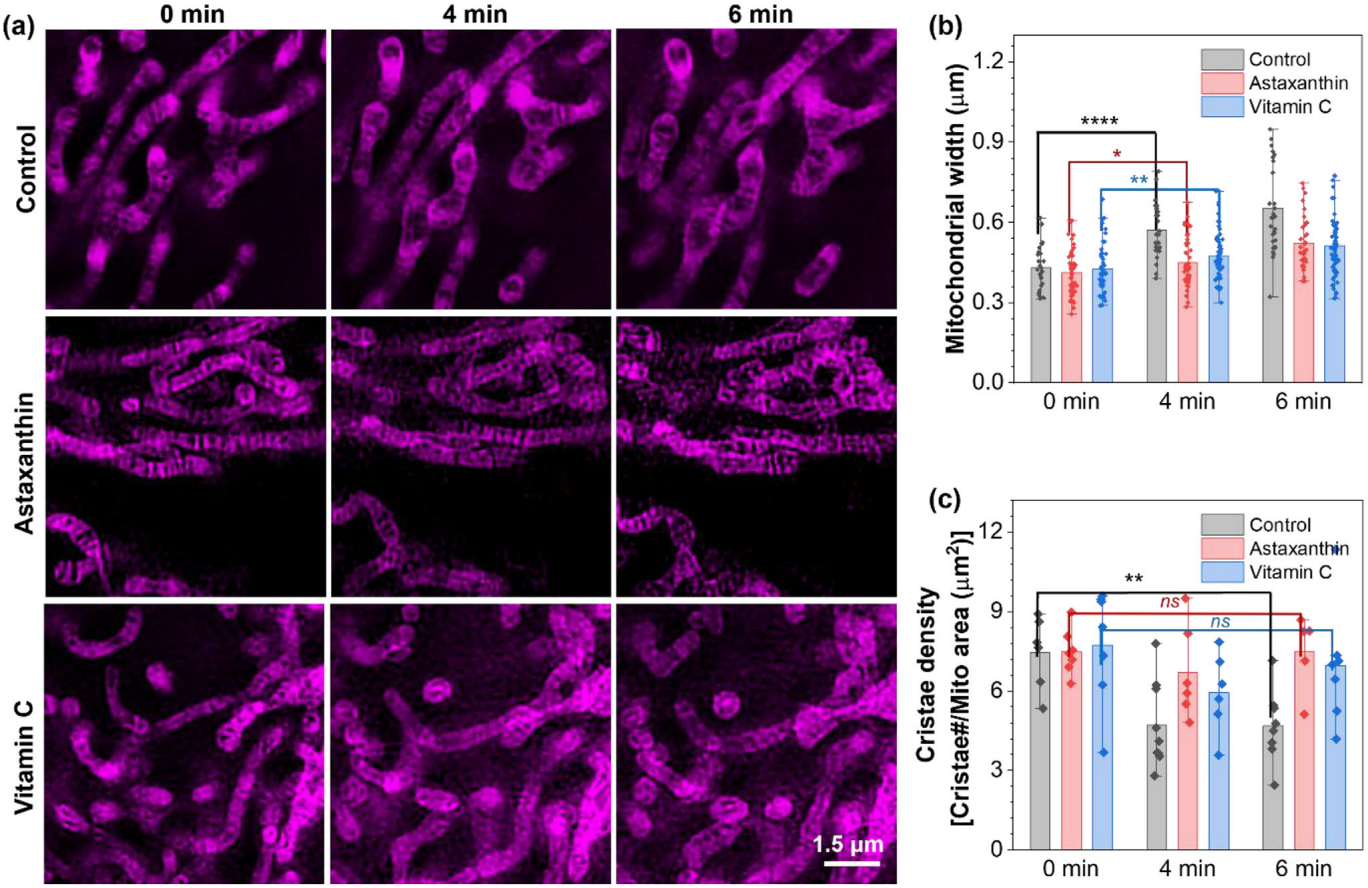
(a) Representative SIM time‐lapse images of OTS‐12C‑stained mitochondria in control, Vitamin C‑treated, and Astaxanthin‑treated cells with continuous light irradiation. (b, c) Quantification of mitochondrial width (b) and cristae density (c) over 6 min of continuous illumination. Data are presented as mean ± SD. Statistical significance was determined via Student's *t*‐test: ^*^ *p*< 0.05; ^**^ *p*< 0.01; ^***^ *p*< 0.001; ^****^ *p*< 0.0001; ns, not statistically significant. The mitigation of cristae disruption by antioxidant pretreatment validates OTS‐12C as a dual‑function probe for imaging and functional assessment of mitochondrial oxidative stress.

We next explored whether this probe could serve as a valuable tool to evaluate the protective effects of antioxidants on mitochondrial ultrastructure. Cells were pretreated with Vitamin C (20 µM) or Astaxanthin (10 µM), followed by staining with OTS‐12C and continuous SIM imaging to induce localized ROS generation. As shown in Figure 6, in control cells (untreated), mitochondrial width increased notably within 4 min of irradiation, accompanied by a progressive decline in cristae density. In contrast, cells treated with either Vitamin C or Astaxanthin maintained stable mitochondrial width and cristae density up to 4 min. With prolonged irradiation, both antioxidant‐treated groups eventually exhibited structural changes, but the extent of width expansion remained significantly smaller than that of the control group throughout the imaging period (Figure S18b). Cristae density was also preserved to a greater extent in antioxidant‐treated cells compared to controls (Figure S18c). These results demonstrate that OTS‐12C enables the visualization of mitochondrial morphology and cristae dynamics, thereby providing a measurable means to assess the protective efficacy of antioxidants. This positions it as a dual‐function probe for both super‐resolution imaging and nanoscale assessment of mitochondrial stress responses.

However, it should be noted that the absolute quantification of ROS generation and its comparison with endogenous mitochondrial ROS levels were not performed in this study. Whether the observed oxidative stress mimics physiological conditions remains to be elucidated. Additionally, while our controls support a ROS‑mediated mechanism, the non‑specific phototoxicity in live‑cell super‑resolution microscopy cannot be fully excluded. Despite these caveats, OTS‑12C provides a unique platform coupling super‑resolution imaging with a controllable oxidative stimulus, offering a valuable complement to conventional mitochondrial probes.

### 2.8 Assessment of Mitochondrial Damage Level by FLIM

**FIGURE 7:**
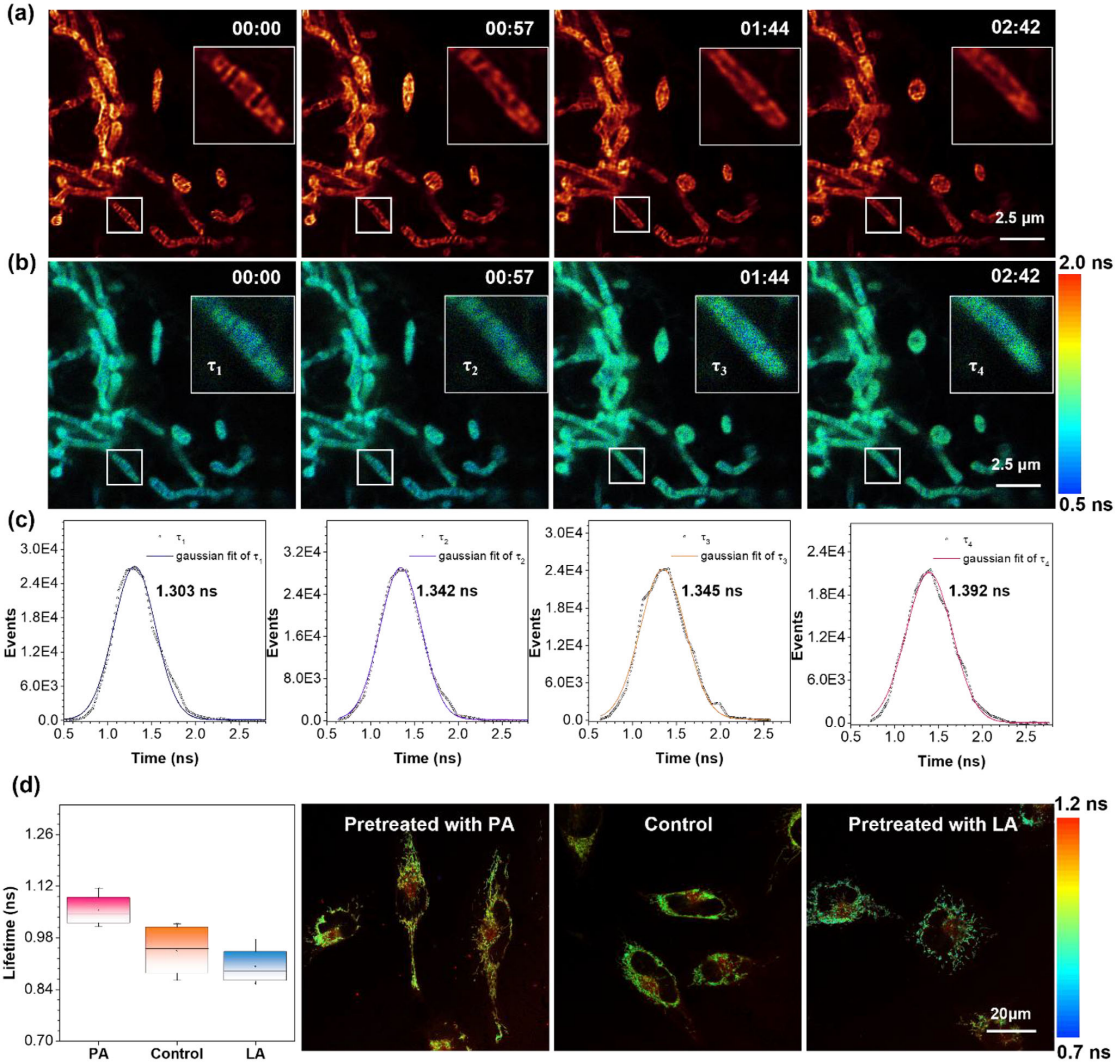
Time‐lapse (a) STED imaging and (b) fluorescence lifetime imaging of mitochondria in living HeLa cells stained with OTS‐12C and (c) the corresponding fluorescence lifetime distribution of OTS‐12C in the enlarged area was analyzed using Gaussian fitting at different time points. (d) Box plots of mean fluorescence lifetime and fluorescence lifetime images of mitochondria labeled by OTS‐12C in living HeLa cells pretreated with or without palmitic acid (PA, 100 µM) and linoleic acid (LA, 400 µM) for 24 h.

To investigate the structural and functional dynamics of mitochondria, we labeled HeLa cells with OTS‐12C and performed time‐lapse imaging using STED‐FLIM microscopy. Initially, mitochondria displayed typical tubular morphology with well‐defined cristae (Figure 7a). During a 3 min imaging period, cristae structures gradually disappeared, while the tubular width of mitochondria remained unchanged, suggesting dynamic remodeling of the IMM rather than cellular damage or dysfunction. Concurrently, we recorded the fluorescence lifetime of OTS‐12C to quantitatively assess changes in the local mitochondrial microenvironment. As shown in Figure 7b,c, within 57 s, the fluorescence lifetime of OTS‐12C increased from 1.303 ns to 1.342 ns, coinciding with the loss of one or two cristae but no apparent change in overall morphology. After 3 min, cristae structures were almost completely absent with the same tubular width of mitochondria, and the fluorescence lifetime further increased to 1.392 ns. During the imaging period, the fluorescence lifetime of OTS‐12C within mitochondria increased without morphological changes, indicating chemical, physical, or mechanical changes in the mitochondria.

To test whether mitochondrial membrane tension influences OTS‐12C fluorescence lifetime, FLIM was performed on HeLa cells before and after osmotic shock, which alters the membrane tension of cells [28]. No significant lifetime changes were observed following osmotic shock (Figure S19), indicating that OTS‐12C lifetime is largely insensitive to membrane tension changes. To test if the OTS‐12C fluorescence lifetime change in mitochondria is related to the mitochondrial membrane potential (MMP) changes, we treated the cells with carbonyl cyanide m‐chlorophenyl hydrazone (CCCP), a protonophore that induces mitochondrial depolarization and decreases MMP. After treatment with CCCP, the increased OTS‐12C lifetime could also be obtained (Figure S20). However, treatment with oligomycin, an ATP synthase inhibitor that increases MMP by blocking proton re‐entry, also increased OTS‐12C lifetime (Figure S21). This suggests that the MMP may not be the main contributor to the fluorescence lifetime changes of OTS‐12C observed, as both increased and decreased MMP would induce fluorescence lifetime elongation. Considering that both CCCP and oligomycin would also induce oxidative stress in mitochondria, leading to oxidation of lipids and alterations in the lipid composition and packing [29], we hypothesized that these lipid changes might play a more significant role in influencing the probe's fluorescence lifetime. To test this hypothesis, we examined the response of the probe to differences in lipid composition and packing. The sensitivity of OTS‐12C lifetime to membrane order was evaluated in large unilamellar vesicles (LUVs) composed of distinct lipid bilayers such as 1,2‐dioleoyl‐sn‐glycero‐3‐phosphocholine (DOPC), sphingomyelin (SM), and cholesterol (Chol). The measurement shows that OTS‐12C lifetime increased in the order DOPC < DOPC/Chol < SM/Chol (Figure S22), demonstrating a correlation between increased membrane order and longer OTS‐12C lifetime. These data suggest that OTS‐12C is sensitive to lipid composition and lipid packing.

Given that ROS‐induced peroxidation of unsaturated lipids in the IMM can increase membrane order [29], the effect of lipid composition on OTS‐12C lifetime was further explored. HeLa cells pretreated with palmitic acid (PA, a saturated fatty acid) exhibited a significantly longer OTS‐12C fluorescence lifetime compared to the control, whereas linoleic acid (LA, a polyunsaturated omega‐6 fatty acid) pretreatment led to a markedly shorter lifetime (Figure 7d). These results imply that increased OTS‐12C lifetime under high‐power laser excitation correlates with ROS‐induced oxidation of unsaturated lipids in the IMM. Collectively, these findings highlight the utility of OTS‐12C as a sensitive probe for assessing changes in IMM lipid composition and membrane order, particularly in the context of oxidative stress. Together, the fluorescence lifetime of OTS‐12C is a sensitive readout of the physicochemical properties of the IMM. Hence, FLIM‐based STED imaging of OTS‐12C provides a robust approach for quantitative assessment of mitochondrial structure and function in live cells at the sub‐organelle level.

## 3 Conclusion

In this study, we present a molecular engineering strategy involving a series of AIE‐active isoquinoline derivatives (OTS‐4C, OTS‐7C, OTS‐12C) bearing carboxyl groups of varying alkyl chain lengths. These probes exhibit markedly reduced phototoxicity compared to their non‐carboxylated counterparts, arising from two synergistic mechanisms: intrinsic suppression of spin‐orbit coupling by the COOH group, and the modulation of AI‐ISC via chain‐length‐dependent molecular packing. Therefore, this molecular design can be used to fine‐tune the phototoxicity of AIE photosensitizers for the development of a functional probe that can be used for super‐resolution imaging under mild and controlled oxidative stress conditions. Both OTS‐7C and OTS‐12C demonstrate specificity for the IMM and robust photostability, facilitating visualization of cristae dynamics using STED and SIM. Rather than focusing solely on resolution, our approach offers a distinct functional capability—the controlled, moderate ROS generation of OTS‐12C serves as a built‐in stimulus to probe mitochondrial structural responses under tunable oxidative stress. Leveraging this feature, we demonstrated that antioxidants such as Vitamin C and Astaxanthin partially preserve cristae architecture under mild ROS stimulation, establishing a new platform for the nanoscale screening of potential drugs that affect mitochondrial function. Time‐lapse imaging with OTS‐12C captured ultrastructural dynamic events, including mitochondrial division, cristae remodeling, and recovery processes. Furthermore, fluorescence lifetime imaging provided a sensitive quantitative readout of sub‐optimal mitochondrial states. Together, these findings establish OTS‐12C as a tool that couples super‐resolution imaging with the functional interrogation of mitochondrial stress responses. This design framework—tuning ROS generation rather than eliminating it entirely—may guide the development of next‐generation probes for studying mitochondrial pathophysiology and evaluating therapeutic candidates in disease models.

## 4 Experimental Section

### 4.1 Materials and Chemicals

All chemicals and reagents were purchased from commercial sources and used as received without further purification. Silver tetrafluoroborate (AgBF_4_) was purchased from TCI. Diphenylacetylene, propylamine, copper (II) acetate [Cu (OAc)_2_], and other chemicals as well as solvents were all purchased from Aldrich. Hydroxyfluorescein (HPF) was purchased from Shanghai Aladdin BioChem Technology Co., Ltd. Vitamin C, 2',7'‐dichlorofluorescin diacetate (DCFH‐DA), 9,10‐anthracenediyl‐bis(methylene) dimalonic acid (ABDA), and rose bengal (RB) were available from Sigma–Aldrich. CCK‐8 kits were purchased from Beyotime. Dulbecco's modified Eagle's medium (DMEM), Dulbecco's phosphate‐buffered saline (DPBS), and fetal bovine serum (FBS) were obtained from Gibco. Milli‐Q water was supplied by a Milli‐Q Plus System (Millipore Corporation, United States). Astaxanthin was donated by BGG AHS Co. Ltd.

### 4.2 Equipment and Methods

UV–vis absorption spectra were measured on a Shimadzu UV‐2600 spectrophotometer, with a medium scanning rate, and quartz cuvettes of 1 cm path length. Photoluminescence spectra were recorded on a Horiba Fluoromax‐4 spectrofluorometer. ^1^H and ^13^C NMR spectra were measured on a Bruker AV 500 NMR spectrometer. High‐resolution mass spectra (HRMS) were recorded on a Bruker Maxis Impact mass spectrometer operated in MALDI‐TOF mode. All density functional theory (DFT) calculations were carried out using the Gaussian 16 package for the geometry optimization and energy calculations based on M062X/6−31G (d, p) with long‐range correction. Morphological analysis was conducted by SEM (Hitachi S‐3400N).

### 4.3 ROS Generation Measurement

The general ROS generation measurements were conducted using DCFH as the indicator, which was converted from 2,7‐dichlorodihydrofluorescein diacetate (DCFH‐DA, 0.5 mL, 1 mM in ethanol) reacting with an aqueous solution of NaOH (2 mL, 1.0 mM) for 30 min at room temperature. The hydrolysate was then neutralized with 7.5 mL PBS buffer solution to get the stock solution with a concentration of 50 µM. PBS buffer solution containing 10 µM DCFH was added to 10 µM OTS derivatives (OTS‐4C, OTS‐7C, and OTS‐12C) (stock solution: 1 mM in dimethylsulfoxide (DMSO)). The irradiation input power was adjusted to 30 mW•cm^−2^ by changing the distance between the lamp and the solution. The fluorescence intensity at 525 nm was recorded to indicate the ROS generation rate.

The •OH generation measurements were conducted using HPF as the indicator. PBS buffer solution containing 5 µM HPF (stock solution: 5 mM in DMF) was added to 10 µM OTS derivatives (stock solution: 1 mM in DMSO). The fluorescence signal of the indicator was monitored in a range of 500–550 nm with the excitation wavelength at 480 nm after the solution was subjected to white light irradiation of 30 mW•cm^−2^. The fluorescence intensity at 514 nm was recorded to indicate the •OH generation rate.

The ^1^O_2_ generation measurements were conducted using ABDA as the indicator. PBS buffer solution containing 10 µM ABDA (stock solution: 10 mM in DMSO) was added to 10 µM OTS derivatives (stock solution: 1 mM in DMSO). The absorption spectra of the indicator were monitored in a range of 330–450 nm with the excitation wavelength at 480 nm after the solution was subjected to white light irradiation of 30 mW•cm^−2^. The absorbance decline relative to the initial value at 380 nm was recorded to indicate the decomposition rates of ABDA (^1^O_2_ generation rate).

### 4.4 Cell Culture

HeLa cells and U2OS cells were cultured in DMEM containing 10% FBS in a 5% CO_2_ humidified incubator at 37°C. Once the cells reached 80%–90% confluence, they were dissociated into single cells with 0.05% trypsin‐EDTA at 37°C for 5 min and passaged at a ratio of 1:4–1:10 in one cell culture dish.

### 4.5 Dark & Light Cytotoxicity Evaluated by CCK‐8 Assay

The cytotoxicity on cells was determined by the standard WST‐8 (2‐(2‐methoxy‐4‐nitrophenyl)‐3‐(4‐nitrophenyl)‐5‐(2,4‐disulfophenyl)‐2H‐tetrazolium, monosodium salt) (CCK‐8) assay. HeLa cells were seeded at a density of 7 × 10^3^ cells per well in standard 96‐well clear microplates with 100 µL of culture medium and cultured overnight to reach 70%–80% confluence. After that, the medium was replaced with 100 µL of fresh medium containing different concentrations of OTS derivatives (0, 1, 5, 10, and 15 µM), and water was used as a vehicle control. After 24 h of incubation, 10 µL of 12 mM CCK‐8 stock solution mixed with 90 µL of the abovementioned OTS derivatives containing fresh medium was added to each well for an additional 1 h of incubation. Under similar steps, cells were treated with OTS‐12C (0, 1, 5, 10, and 15 µM) for 1 h, after which the probe‐containing medium was removed, the cells were washed twice with fresh medium, and then incubated in probe‐free medium for an additional 24 h before the CCK‐8 assay. The absorbance at 450 nm (OD450) was measured using the BMG CLARIOstar Microplate Reader. Cell viability (%) was calculated: (OD450 sample/OD450 control) × 100%. For the light cytotoxicity assay, after incubation with different concentrations of OTS derivatives solutions for 1 h, cells were irradiated under LED light source at 30 mW•cm^−2^ for 5, 10, 15, and 20 min, respectively, and continued to culture until a total time of 24 h. All the other procedures were the same as dark cytotoxicity.

### 4.6 Evaluation of Photostability

The confocal images were recorded on a Leica TCS SP8 system. The confocal images of HeLa cells labeled with OTS‐7C, OTS‐12C, and MitoTracker Green FM were repeatedly recorded using Adaptive Focus Control (AFC) to maintain the focus during time‐lapse imaging. The imaging conditions of these probes were identical (under 488 nm excitation at 0.1 mW laser power). The total signal intensity of each image was measured with ImageJ, normalized to the value of the first image, and plotted as a function of the number of recorded confocal images.

### 4.7 Colocalization Assays

HeLa cells were seeded in glass bottom dishes and subjected to subsequent experiments at a cell density of 70%–80%. Hela cells were stained with 5 µM OTS derivatives (OTS‐4C, OTS‐7C, and OTS‐12C) for 30 min and 100 nM commercial organelle markers (MitoTracker Green FM, CellMask Deep Red, ER Tracker Green, LysoTracker Deep Red) for 15 min. The cells were then washed with PBS (3 × 1 mL per dish), and 1 mL of medium was added to each dish for imaging assay. Confocal images were acquired using the Leica TCS SP 8 system. The incubated cells were excited at 488 nm for MitoTracker Green FM and ER‐Tracker Green, and 640 nm for CellMask Deep Red and LysoTracker Deep Red with semiconductor lasers, and the emission signals were collected at 520 ± 20 nm for MitoTracker Green FM and ER‐Tracker Green, 590 ± 40 nm for OTS derivatives, and 670 ± 20 nm for CellMask Deep Red and LysoTracker Deep Red, respectively.

### 4.8 Confocal / STED Imaging of OTS Derivatives Labeled HeLa Cells

HeLa cells were seeded in glass bottom dishes and subjected to subsequent experiments at a cell density of 70%–80%. HeLa cells were stained with 5 µM OTS derivatives for 30 min. The cells were then washed with PBS (3 × 1 mL per dish), and 1 mL of medium was added to each dish for imaging assay. Confocal images were acquired using a Leica Stellaris 8 STED system. The stained cells were excited at 488 nm, and the emission signals were collected at 600 ± 50 nm. A 775 nm STED laser was used at 15 mW at the sample.

### 4.9 Long‐Term Dynamic SIM Super‐Resolution Imaging of Mitochondria Under Stress

The cells were first incubated with 5 µM OTS‐12C for 30 min. After washing three times with PBS, the cells were imaged using SIM. The imaging conditions for the HIS‐SIM (High Intelligent and Sensitive SIM) microscope were set as follows: laser power at 2 or 6.4 mW, exposure time at 10 ms, and continuous acquisition without intervals.

### 4.10 Long‐Term Dynamic SIM Super‐Resolution Imaging of Mitochondria After Antioxidant Treatment

The cells were pretreated with 10 µM Astaxanthin or 20 µM Vitamin C, followed by incubation with 5 µM OTS‐12C for 30 min. Subsequently, the cells were imaged using SIM. The imaging conditions for the HIS‐SIM microscope were set as follows: laser power at 3.5 mW, exposure time at 10 ms, and an interval time of 30 s.

### 4.11 Quantification of Mitochondrial Cristae Density and Architecture

Quantification of mitochondrial cristae was performed following the machine‑learning‑based protocol described by Segawa et al. (2020) using the Trainable Weka Segmentation (TWS) plugin in Fiji (ImageJ) [30]. All analyses were conducted on raw or background‑subtracted super‑resolution images (SIM) of live cells stained with OTS‑12C.

### 4.12 FLIM Imaging of Stimulated Cells

#### 4.12.1 Photo‐Irradiation

A FLIM image of HeLa cells labeled with OTS‐12C was taken before starting irradiation. The cells in the same area were then subjected to photo‐irradiation by an intense laser (WLL, 488 nm) on a Leica Stellaris 8 STED system, and the photo‐irradiation was continued until mitochondria were adequately swollen. The FLIM image was captured under the same acquisition conditions.

#### 4.12.2 Modulation of Phospholipids in the IMM

HeLa cells pre‐treated with linoleic acid (400 µM) or palmitic acid (100 µM) for 24 h were incubated in cellular medium containing OTS‐12C (5 µM) for 30 min. After washing, FLIM images were recorded.

#### 4.12.3 Chemical Treatment

Before being treated with OTS‐12C for 30 min, HeLa cells were incubated with DMEM containing 10 µM oligomycin for 1 h, or 10 µM CCCP for 3 h at 37°C in a 5% CO_2_ incubator.

### 4.13 Statistical Analysis

Statistical analyses and *p*‐values were computed using Origin 2021b. The statistical differences between experimental and control groups were analyzed by *t*‐test analysis. The number of mitochondrial cristae was quantified using ImageJ.

## Funding

This work was partially supported by the National Natural Science Foundation of China (Grant 82422040), the Start‐up Grant from Vice‐Chancellor Early Career Professorship Scheme, The Chinese University of Hong Kong (4933623), Collaborative Research Impact Matching Scheme (CRIMS), Faculty of Science, The Chinese University of Hong Kong, and the Research Grants Council of the Hong Kong Special Administrative Region, China (AoE/M‐402/25‐N).

## Conflicts of Interest

The authors declare no conflict of interest.

## Supporting information




**Supporting File 1**: advs76013‐sup‐0001‐SuppMat.docx.

**Supporting File 2**: advs76013‐sup‐0002‐Data1.pptx.

## Data Availability

The data that support the findings of this study are available from the corresponding author upon reasonable request.
