# Supplementary material for: A Molecular Engineering Strategy to Fine‐Tune Phototoxicity of AIE Probes for Super‐Resolution Imaging of Mitochondrial Cristae Dynamics
Source: Adv Sci (Weinh). 2026 Jul 6:e24281. Online ahead of print. doi: 10.1002/advs.202524281 (PMC13335114; doi:10.1002/advs.202524281)
Supplement: Supplementary file 1 — Supporting File 1: advs76013‐sup‐0001‐SuppMat.docx. [file ADVS-9999-e24281-s002.docx]

Supporting Information

A Molecular Engineering Strategy to Fine-Tune Phototoxicity of AIE Probes for Super-Resolution Imaging of Mitochondrial Cristae Dynamics

Kongqi Chen, Mengjie Wang, Chuen Kam, Zhiming Wang, Qinrong Zhang, Sijie Chen,*

**1. Experimental Procedures**

1.1 **Synthesis**

**Scheme 1** Synthesis of OTS-4C, OTS-7C, and OTS-12C.

**OTS-12C**: Compound 1 was synthesized according to the literature reported before. ^[1]^ A mixture of [RhCp*Cl_2_]_2_ (2.0 mol %), AgBF_4_ (0.30 mmol), Cu (OAc)_2_ (0.30 mmol), Compound 1 (0.30 mmol), Diphenylacetylene (0.36 mmol), and 4-Aminobutyric acid (0.45 mmol) in 2.5 mL of tert-amyl alcohol were heated and stirred under nitrogen at 110°C for 3 h. When the reaction was complete, the mixture was cooled and diluted with CH_2_Cl_2_ (10 mL). The mixture was filtered through a Celite pad, and the Celite pad was washed with CH_2_Cl_2_ (30 mL) and MeOH (20 mL). The residues were purified by silica column chromatography using CH_2_Cl_2_/MeOH (100:1, v/v) as eluent to give pure products. The solids were further precipitated from CH_2_Cl_2_/PE mixtures under sonication and filtered to obtain powder-like products. ^1^H NMR (500 MHz, DMSO) δ 9.85 (s, 1H), 7.74 (d, *J* = 8.7 Hz, 2H), 7.43 (d, *J* = 26.6 Hz, 5H), 7.27 (dd, *J* = 23.0, 7.3 Hz, 6H), 7.14 (d, *J* = 8.6 Hz, 4H), 6.98 (d, *J* = 8.7 Hz, 4H), 6.73 (d, *J* = 8.7 Hz, 2H), 4.26 (m, 2H), 3.76 (s, 6H), 2.17 (t, *J* = 7.2 Hz, 2H), 1.76 (m, 2H), 1.47 (m, 2H), 1.25 – 1.01 (m, 14H).^13^C NMR (126 MHz, DMSO) δ 157.37, 138.87, 130.76, 130.16, 128.82, 128.41, 117.54, 115.69, 55.80, 29.22, 29.03, 28.41, 25.87, 25.05. HRMS (ESI): m/z (cation) 789.3690 [M^+^, calcd 789.3720].

**OTS-7C**: This compound was prepared similarly as described for OTS-4C. Red solid, yield: 78%.^1^H NMR (500 MHz, DMSO) δ 9.89 (s, 1H), 7.74 (d, *J* = 8.6 Hz, 2H), 7.43 (d, *J* = 27.1 Hz, 5H), 7.35 – 7.22 (m, 6H), 7.15 (d, *J* = 8.7 Hz, 4H), 6.98 (d, *J* = 8.7 Hz, 4H), 6.73 (d, *J* = 8.6 Hz, 2H), 4.27 (m, 2H), 3.76 (s, 6H), 1.99 (m, 2H), 1.76 (m, 2H), 1.35 (m, 2H), 1.15 – 1.05 (m, 4H). ^13^C NMR (126 MHz, DMSO) δ 157.37, 138.87, 130.17, 129.42, 128.84, 128.40, 117.54, 115.69, 55.80, 30.57, 29.47. HRMS (ESI): m/z (cation) 719.2912 [M^+^, calcd 719.2938].

**OTS-4C**: This compound was prepared similarly as described for OTS-4C. ^1^H NMR(500 MHz, DMSO) δ 9.97 (s, 1H), 7.66 (s, 2H), 7.43 (s, 2H), 7.38 (d, *J* = 6.6 Hz, 3H), 7.29 (s, 3H), 7.22 (d, *J* = 6.9 Hz, 3H), 7.11 (d, *J* = 8.5 Hz, 4H), 6.97 (d, *J* = 8.7 Hz, 4H), 6.70 (d, *J* = 8.2 Hz, 2H), 4.34 (m, 2H), 3.76 (s, 6H), 1.89 (m, 4H). ^13^C NMR (126 MHz, DMSO) δ 157.36, 138.83, 134.21, 130.75, 130.14, 128.77, 128.37, 117.51, 115.68, 55.79, 27.72. HRMS (ESI): m/z (cation) 677.2443 [M^+^, calcd 677.2468].

**2. Figures**


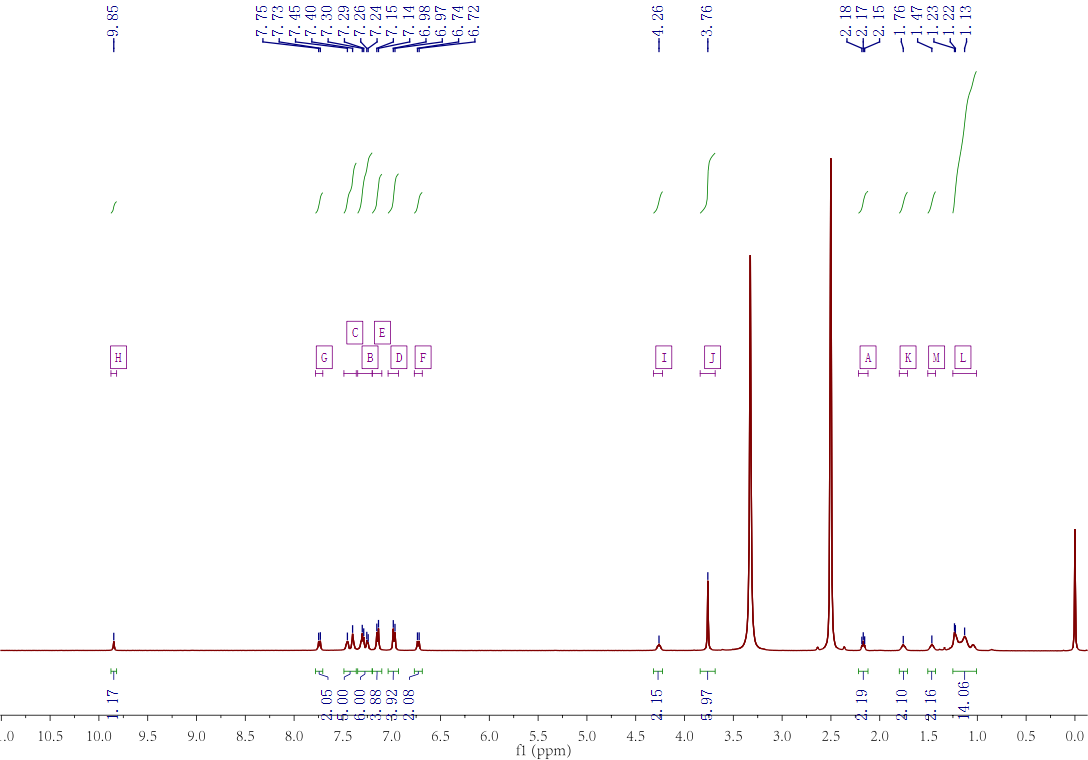


**Figure S1.** ^1^H NMR spectrum of OTS-12C in DMSO-*d6*.


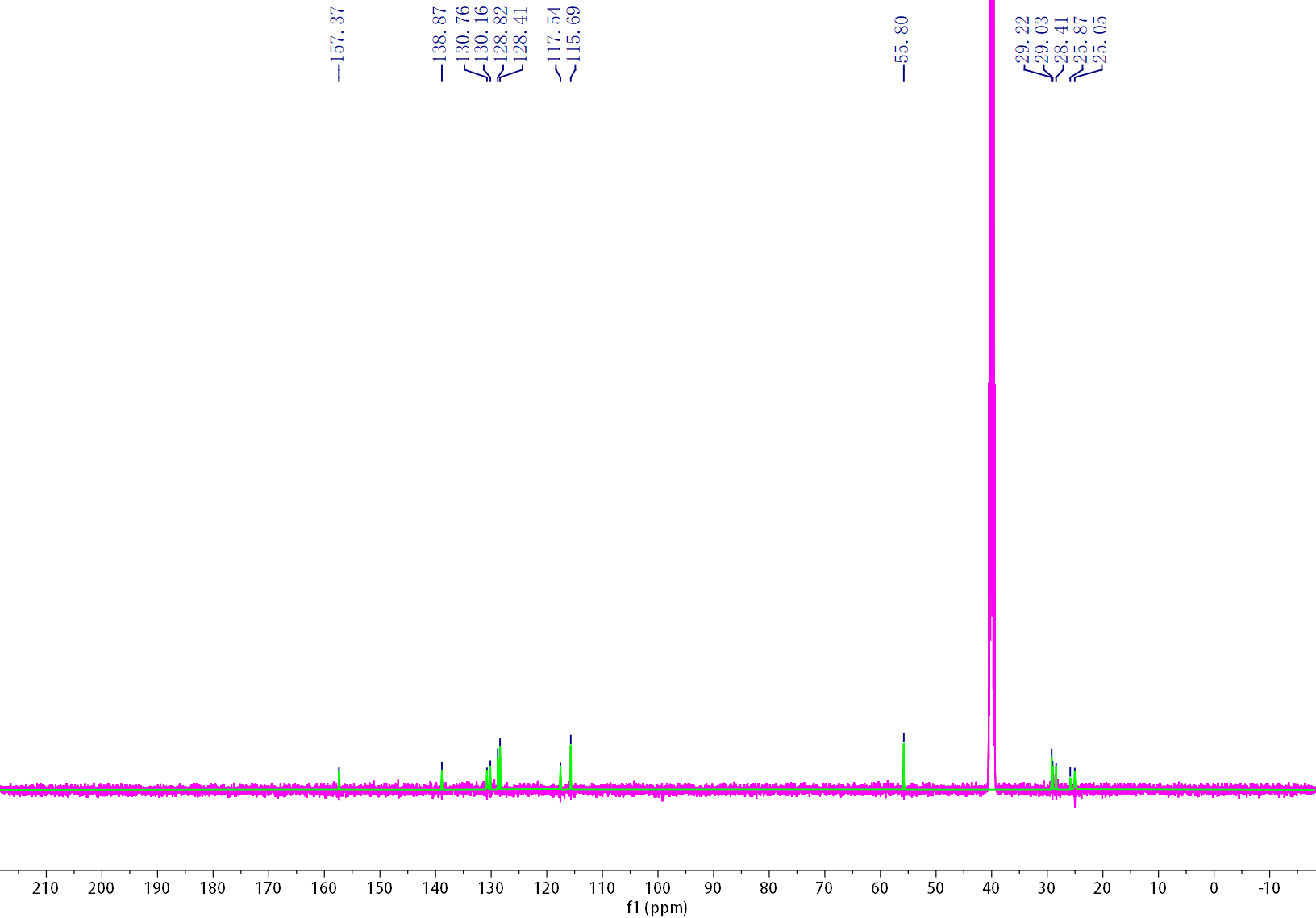


**Figure S2.** ^13^C NMR spectrum of OTS-12C in DMSO-*d6*.


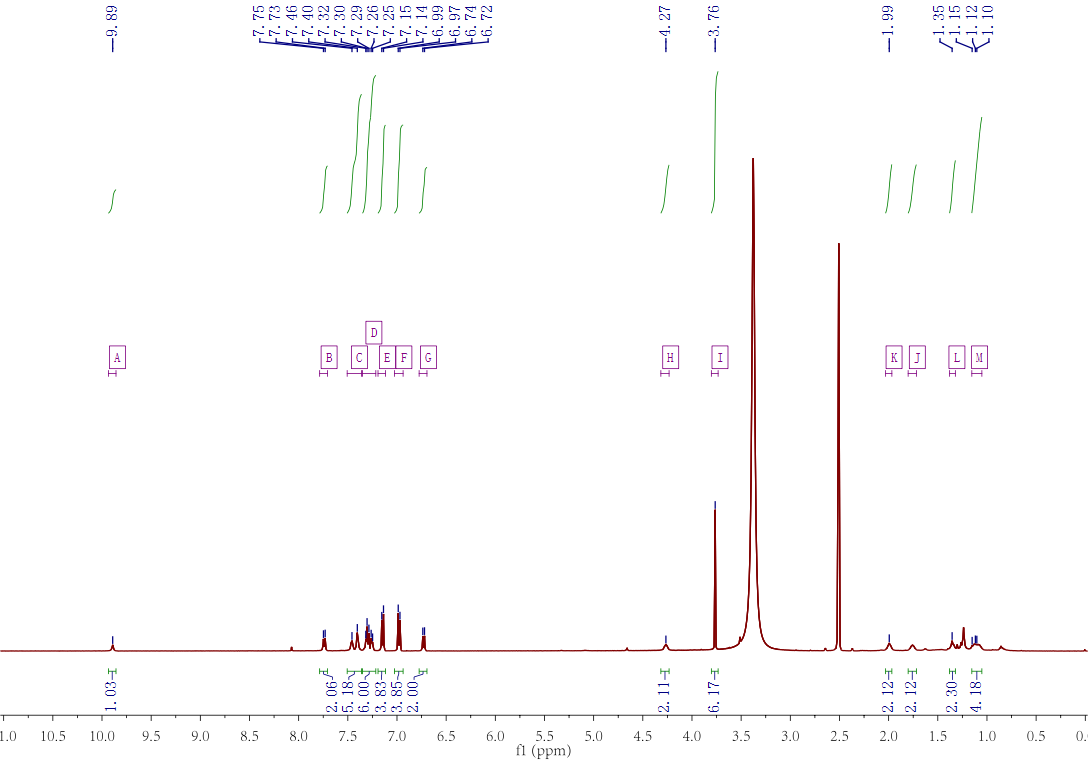


**Figure S3.** ^1^H NMR spectrum of OTS-7C in DMSO-*d6*.


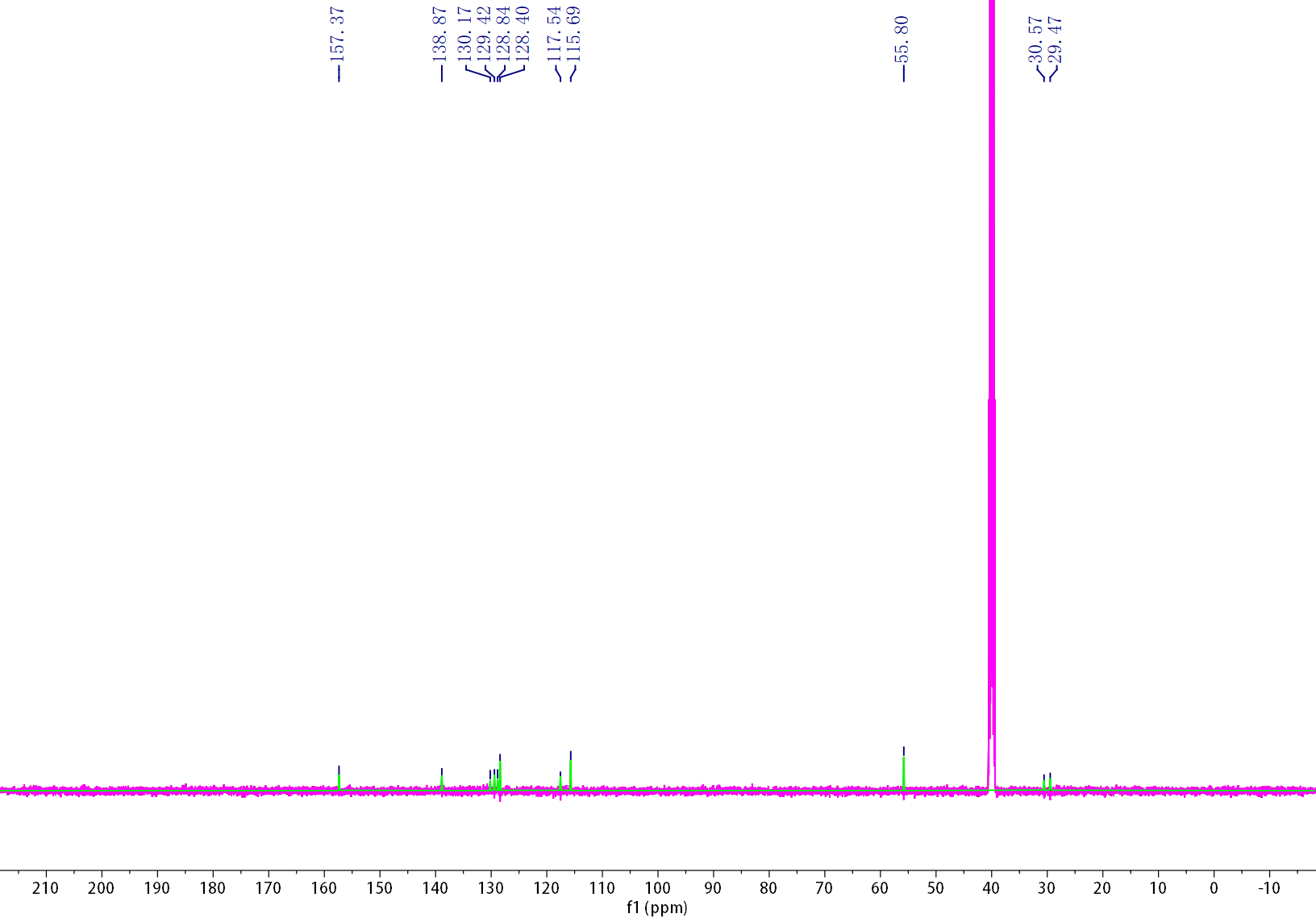


**Figure S4.** ^13^C NMR spectrum of OTS-7C in DMSO-*d6*.


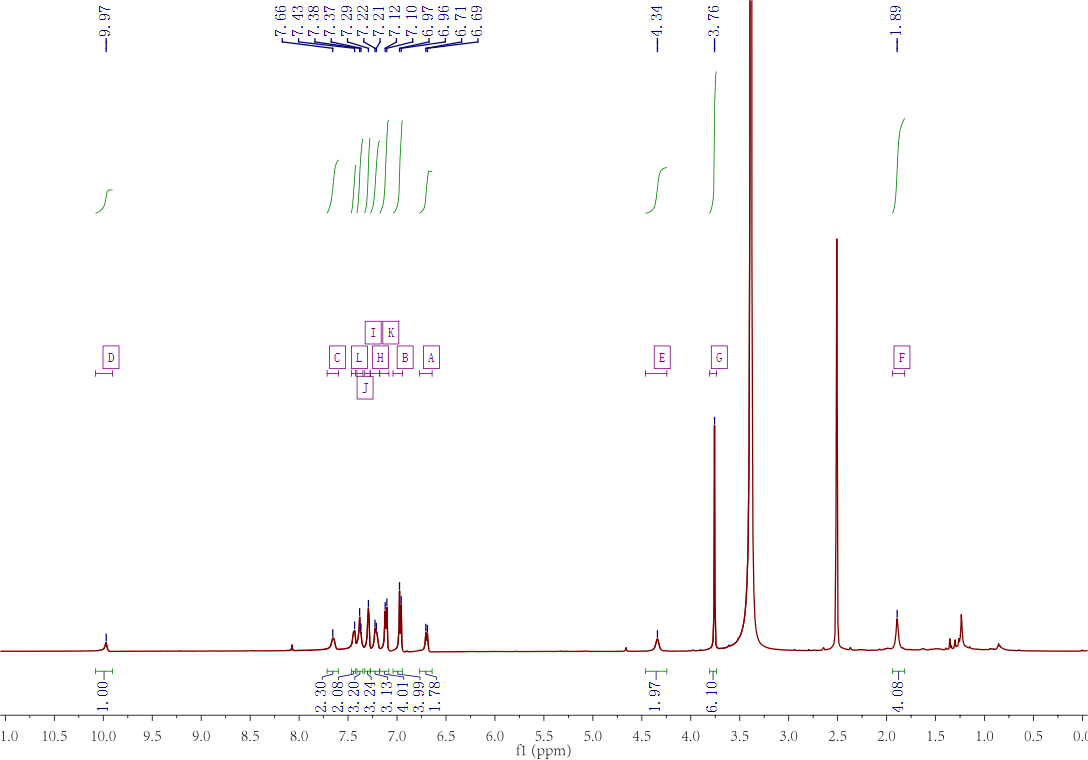


**Figure S5.** ^1^H NMR spectrum of OTS-4C in DMSO-*d6*.


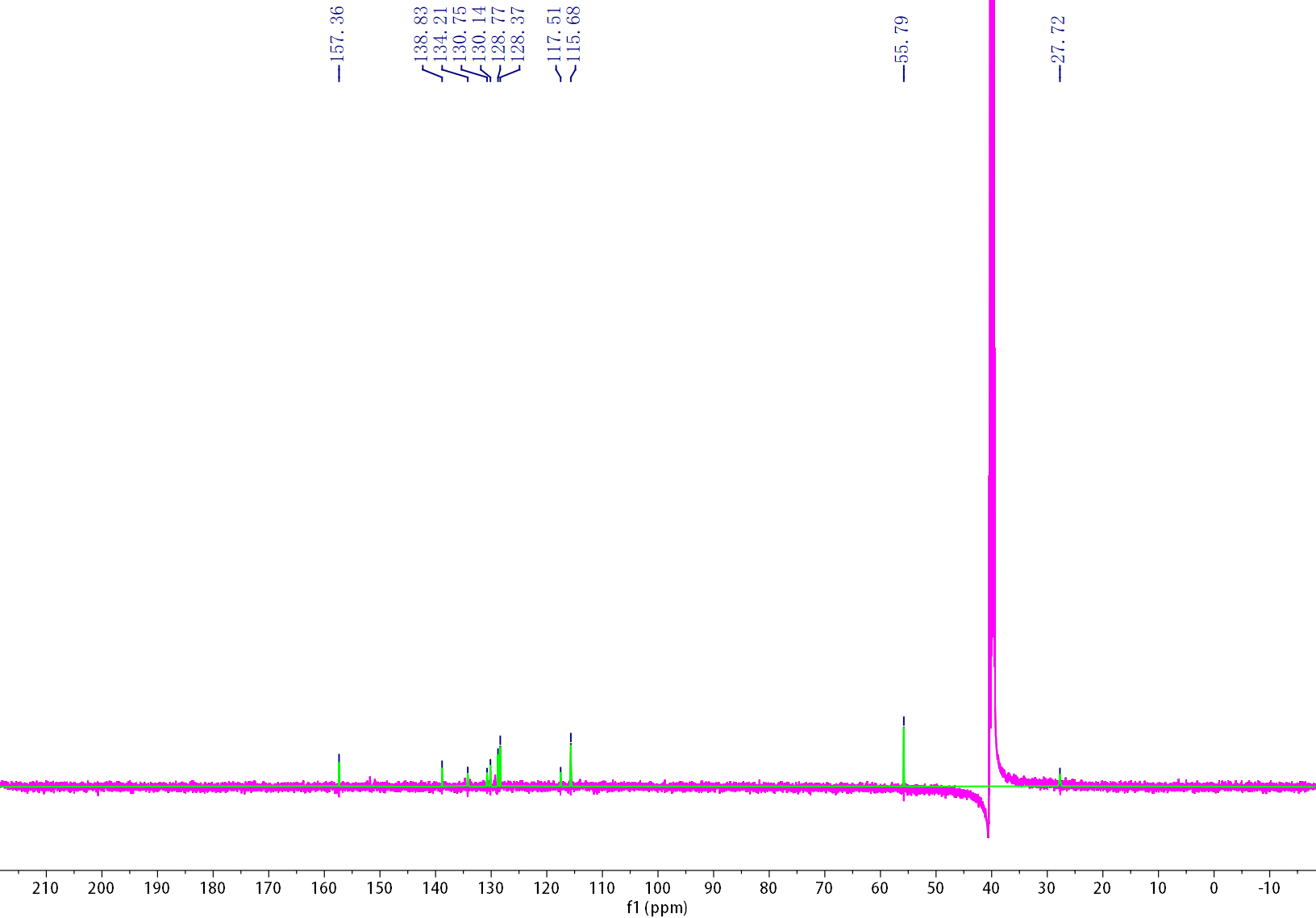


**Figure S6.** ^13^C NMR spectrum of OTS-4C in DMSO-*d6*.


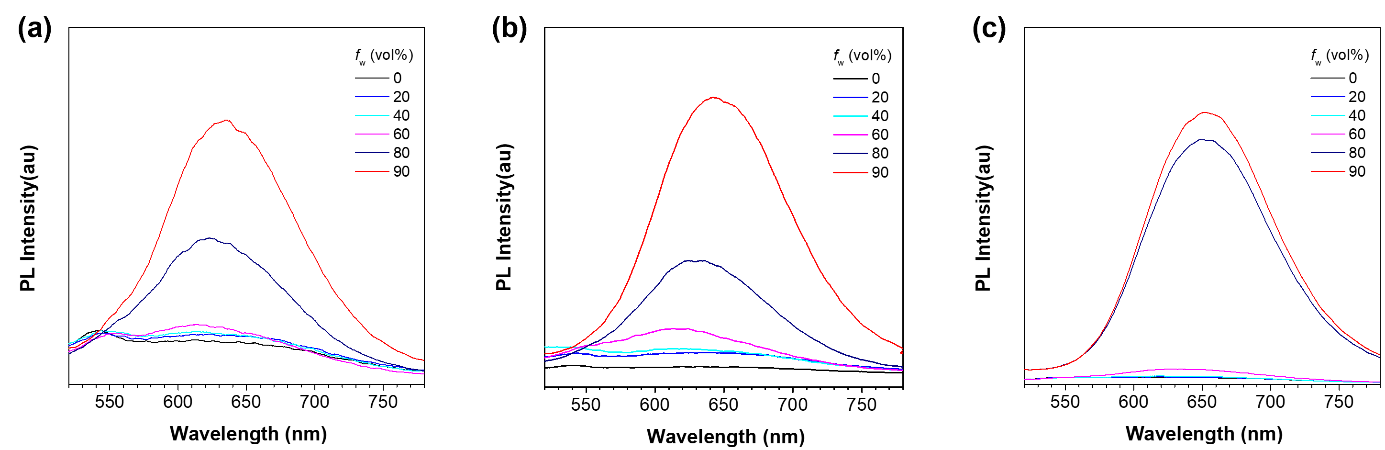


**Figure S7.** Photoluminescence (PL) spectra of OTS-4C (a), OTS-7C (b), and OTS-12C (c) in DMSO/water mixtures with different water fractions (*f*_w_) under the same excitation conditions.


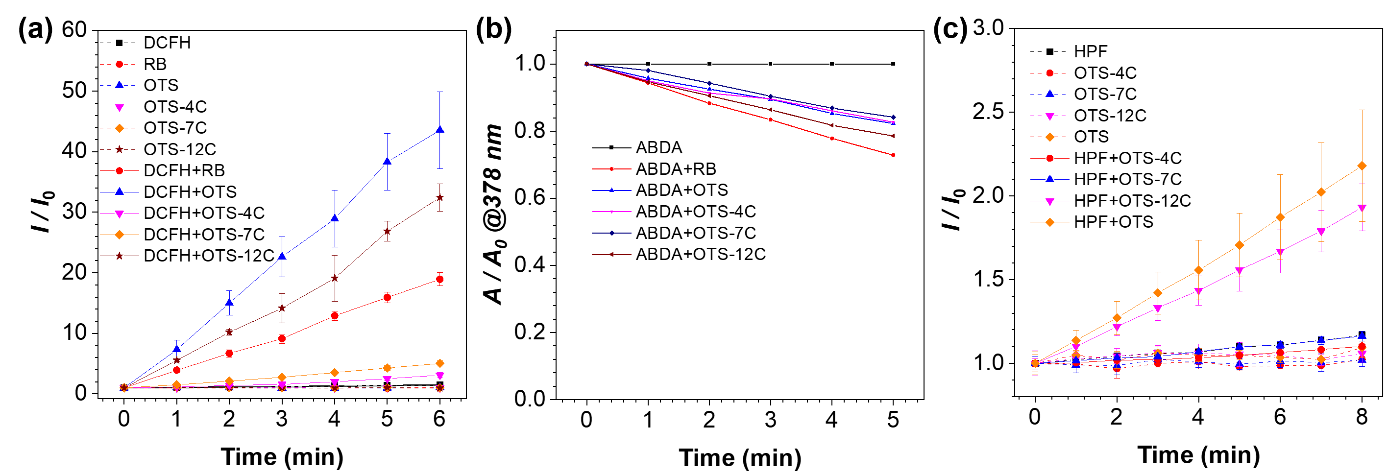


**Figure S8.** (a) Plots of the relative PL intensity of DCFH (for general ROS detection) and (b) Plots of decomposition rates of ABDA (for ^1^O_2_ detection), and (c) plots of relative PL intensity of HPF (for •OH detection) in the presence of 10 μM OTS, OTS-4C, OTS-7C, and OTS-12C in PBS with 1 vol % DMSO versus irradiation time (white light, 30 mW•cm^–2^).


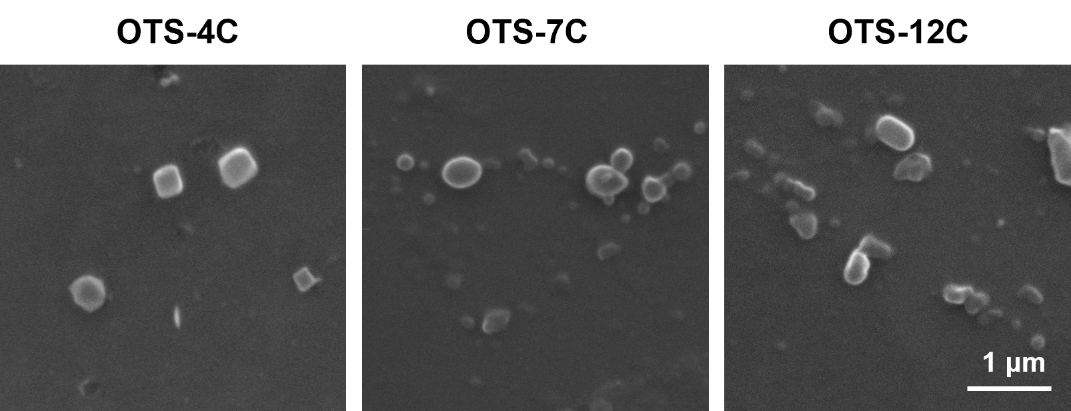


**Figure S9.** SEM images of kiln-dried films from the aqueous solution of OTS‑4C, OTS‑7C, and OTS‑12C. Scale bars: 1 µm.


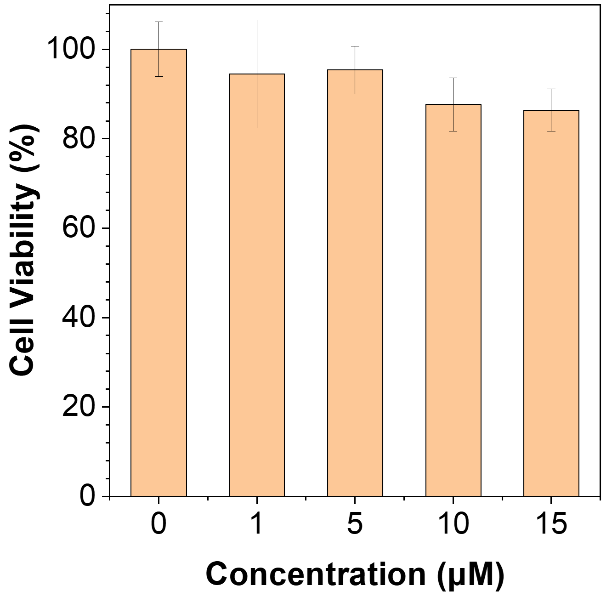


**Figure S10.** Cell viability of HeLa cells incubated with varied concentrations of OTS-12C for 1 h followed by 24 h culture.


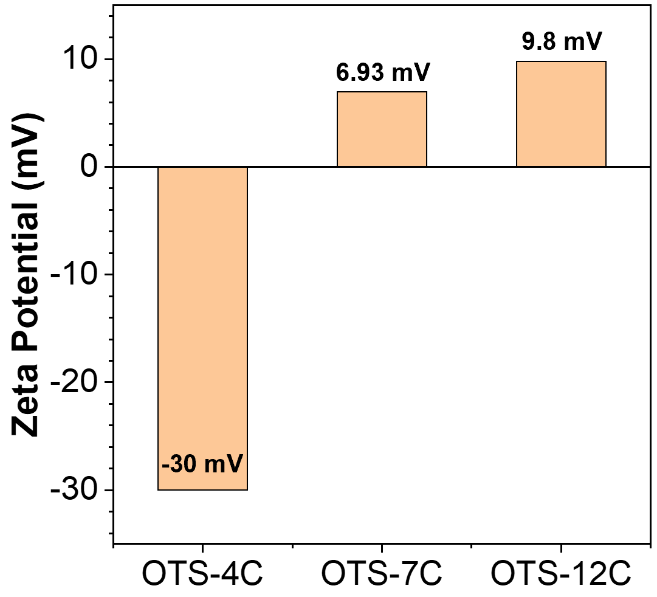


**Figure S11.** Zeta potentials of OTS-4C, OTS-7C, and OTS-12C nanoparticles.


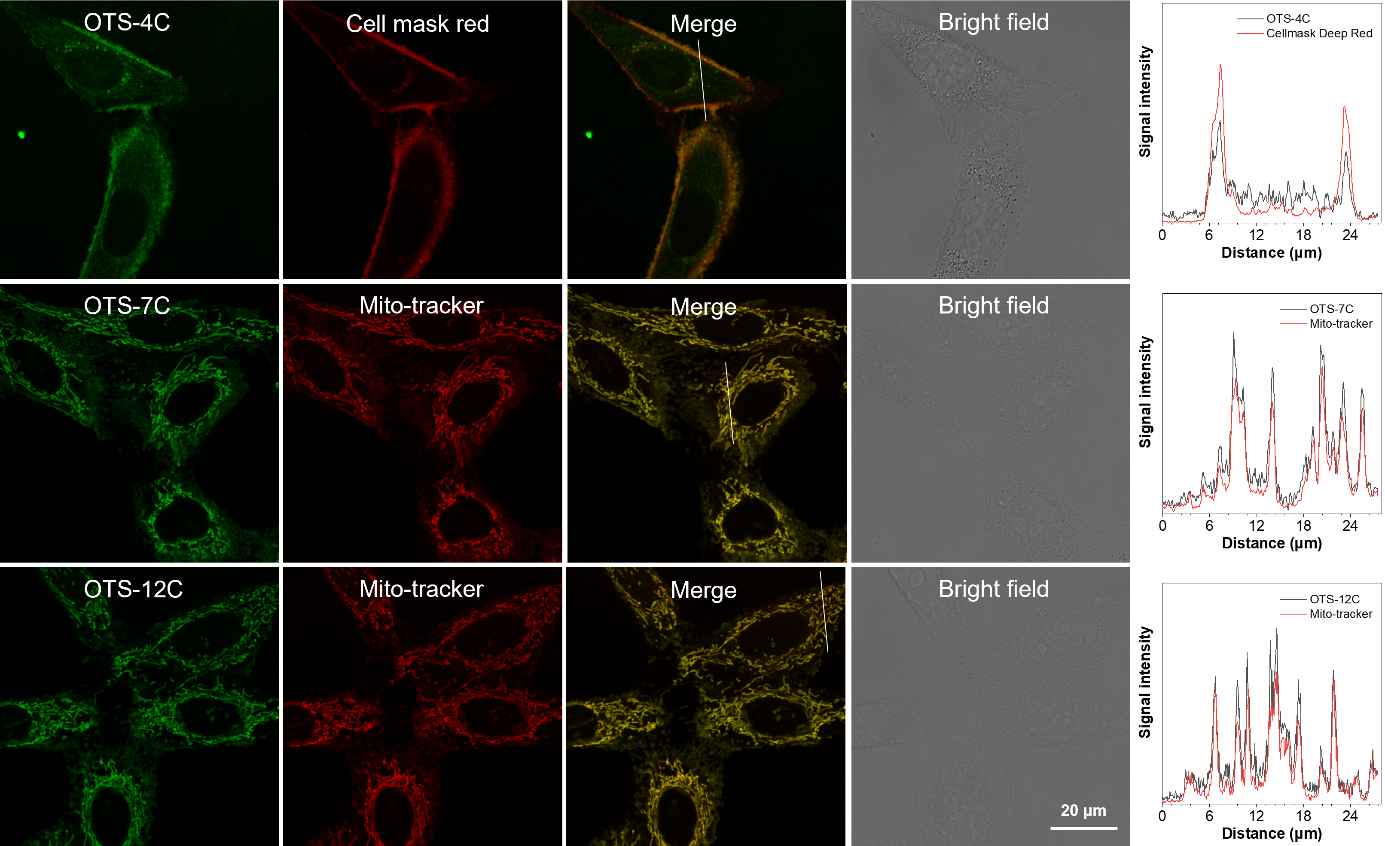


**Figure S12.** Colocalization images of HeLa cells stained with OTS-4C, OTS-7C, and OTS-12C and MitoTracker or CellMask Deep Red. All the images share the same scale bar; scale bar: 20 µm.


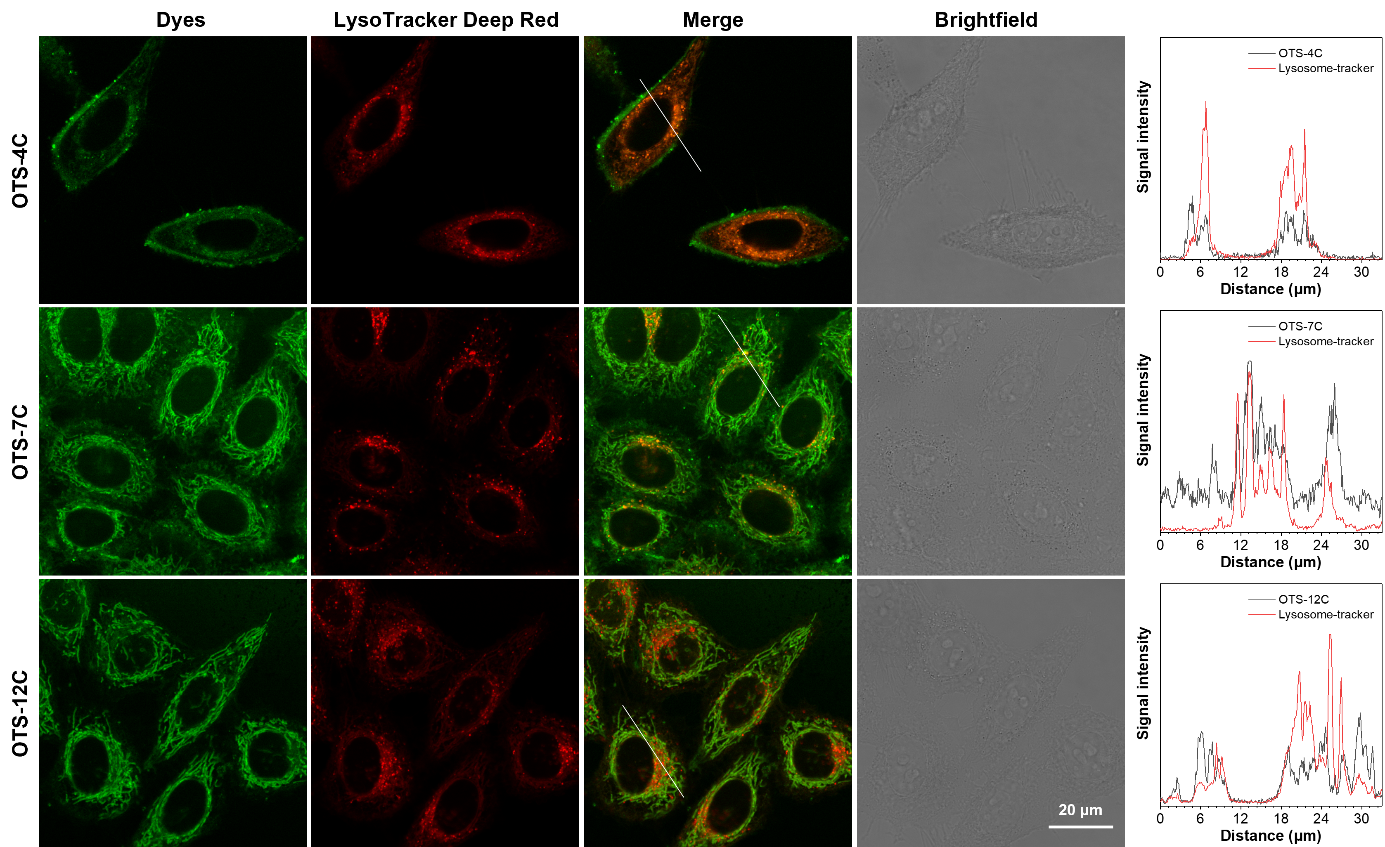


**Figure S13.** Fluorescence and brightfield images of HeLa cells co-stained with OTS derivatives and LysoTracker Deep Red. Fluorescence images are pseudocolored. The corresponding line plot analysis along the white line in the panel shows a poor correlation between OTS derivatives and LysoTracker Deep Red fluorescence signals.


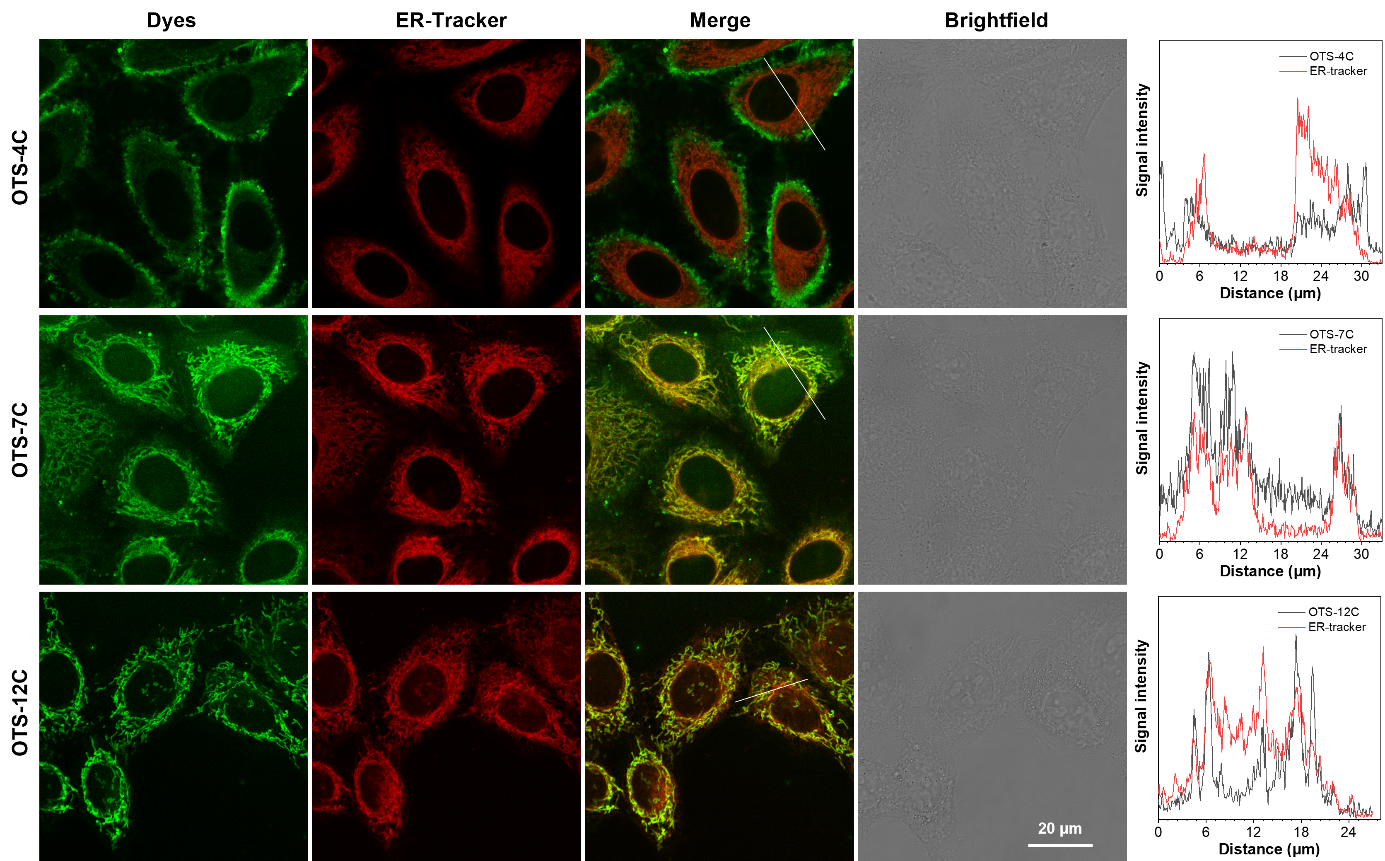


**Figure S14.** Fluorescence and brightfield images of HeLa cells co-stained with OTS derivatives and ER-Tracker. Fluorescence images are pseudocolored. The corresponding line plot analysis along the white line in the panel shows a poor correlation between OTS derivatives and ER-Tracker fluorescence signals.


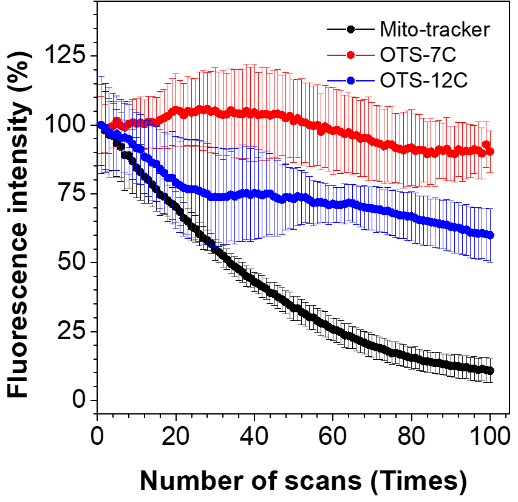


**Figure S15.** The photostability of MitoTracker Green, OTS-7C, or OTS-12C was tested over 10 minutes using a confocal laser scanning microscope.


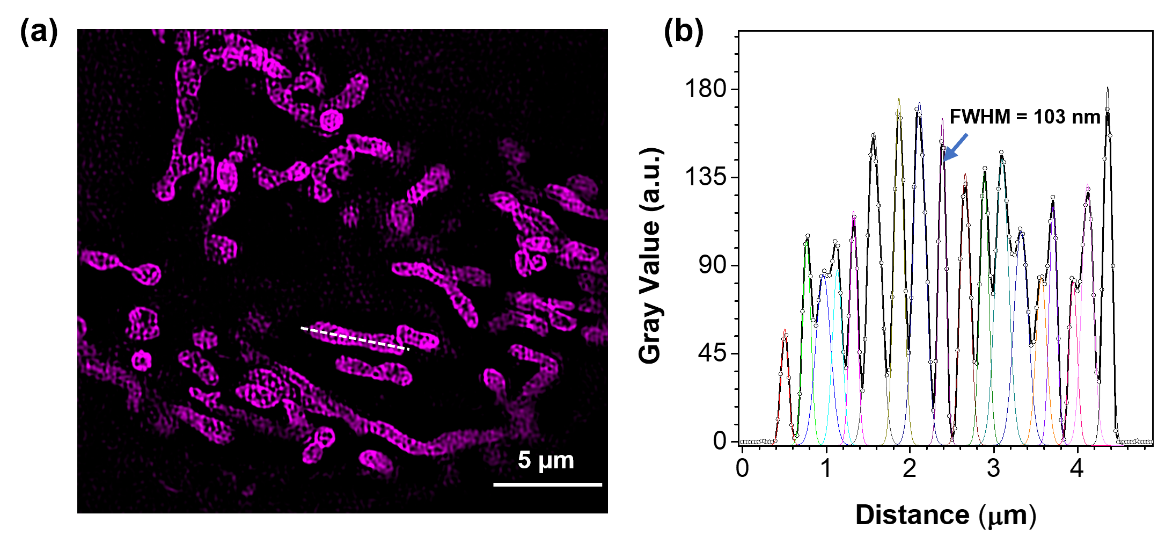


**Figure S16.** (a) SR imaging of mitochondria in HeLa cells using Hessian-SIM and (b) the corresponding intensity profiles along the lines across cristae in HeLa cells stained with OTS-12C.


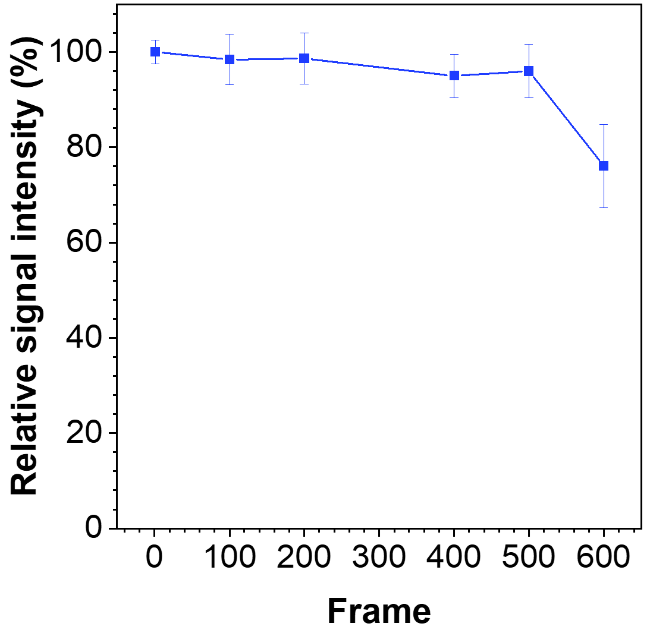


**Figure S17.** Quantitative analysis of normalized fluorescence intensity over 200 consecutive frames (*n* = 5 cells, mean ± SEM) from Figure 5a.


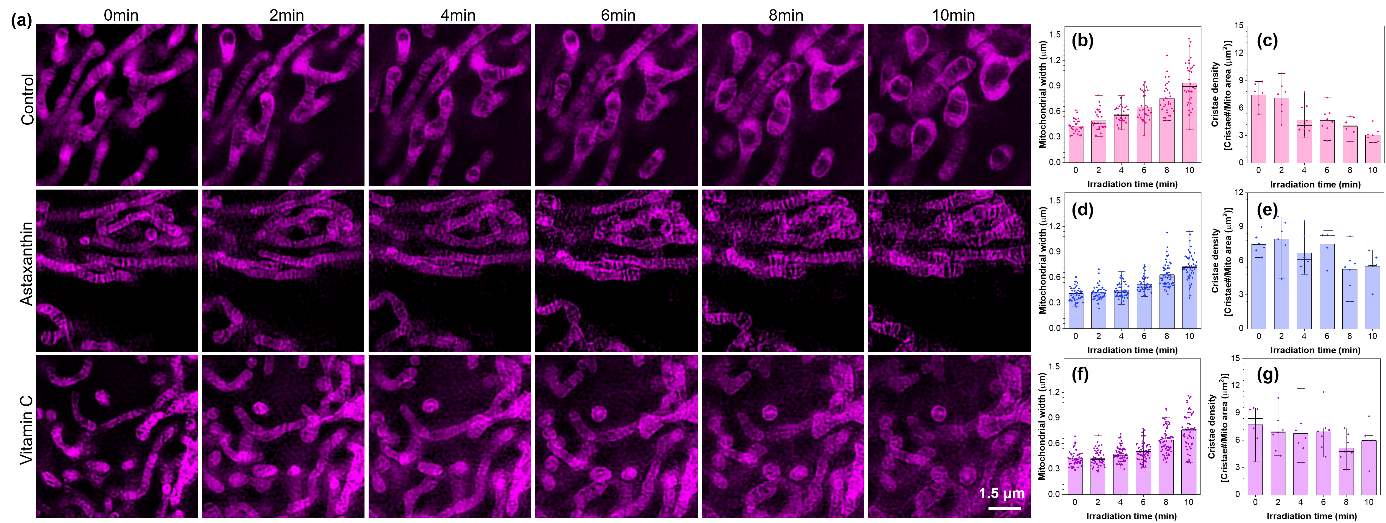


**Figure S18.** (a) SIM time-lapse images of OTS-12C‑stained mitochondria in control, Astaxanthin‑treated, and Vitamin C‑treated cells with continuous light irradiation. (b-g) Quantification of mitochondrial width (b, d and f) and cristae density (c, e and g) over 10 min of continuous illumination of OTS-12C‑stained mitochondria in control (b and c), Astaxanthin ‑treated (d and e), and Vitamin C‑treated (f and g) cells.


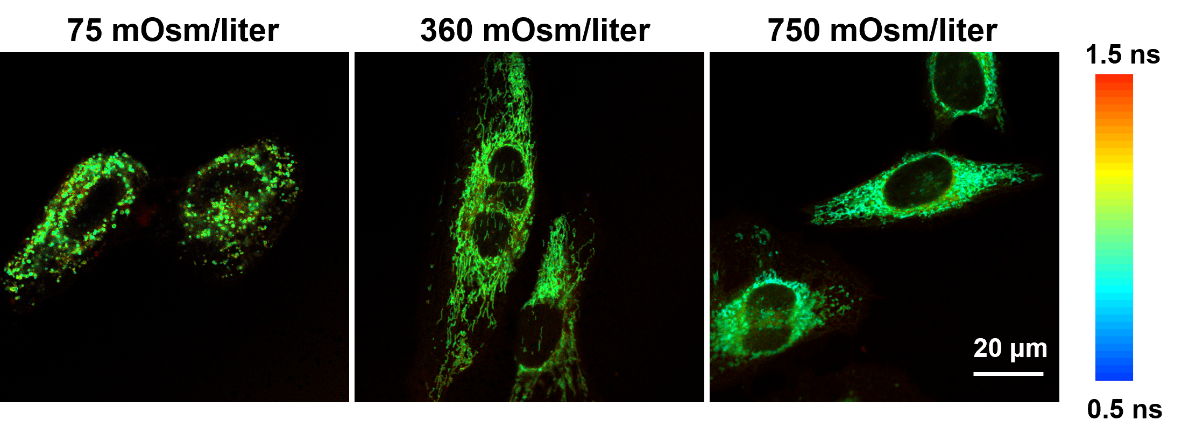


**Figure S19.** The fluorescence lifetime images of OTS-12C in HeLa cells under the hypoosmotic condition (75 mOsm L^–1^), the isosmotic condition (360 mOsm L^–1^), and the hyperosmotic condition (750 mOsm L^–1^).


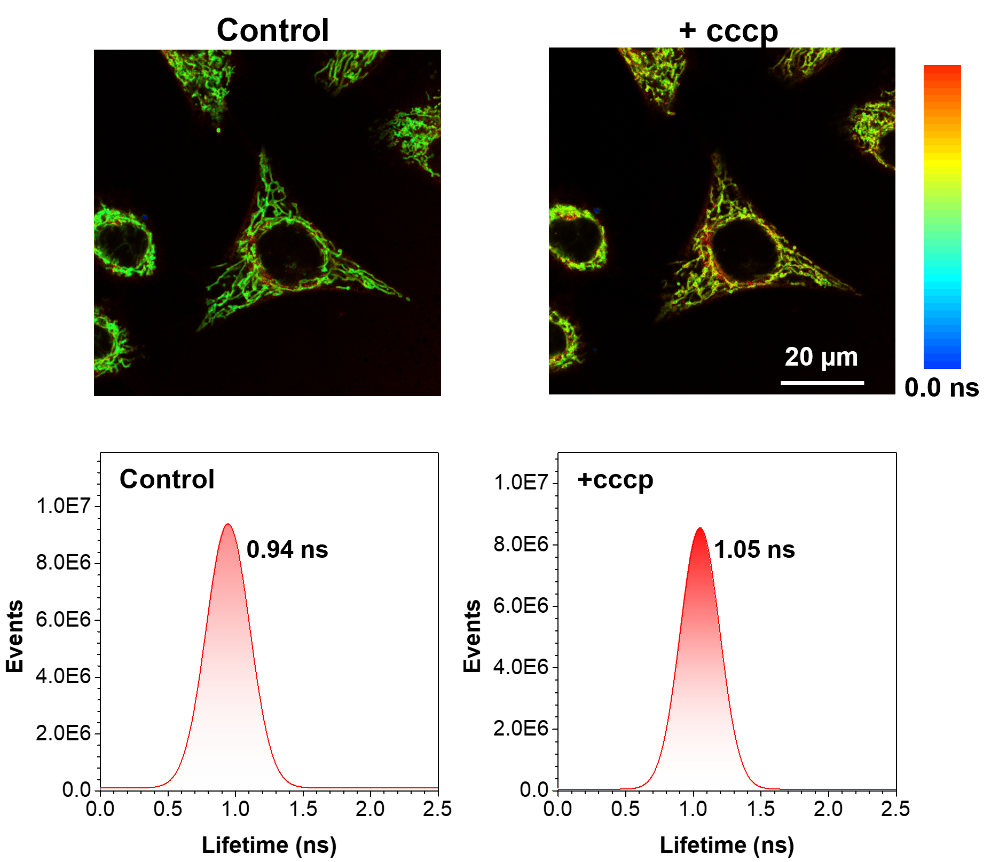


**Figure S20.** FLIM images and fluorescence lifetime histograms of HeLa cells stained with OTS-12C treatment with or without CCCP.


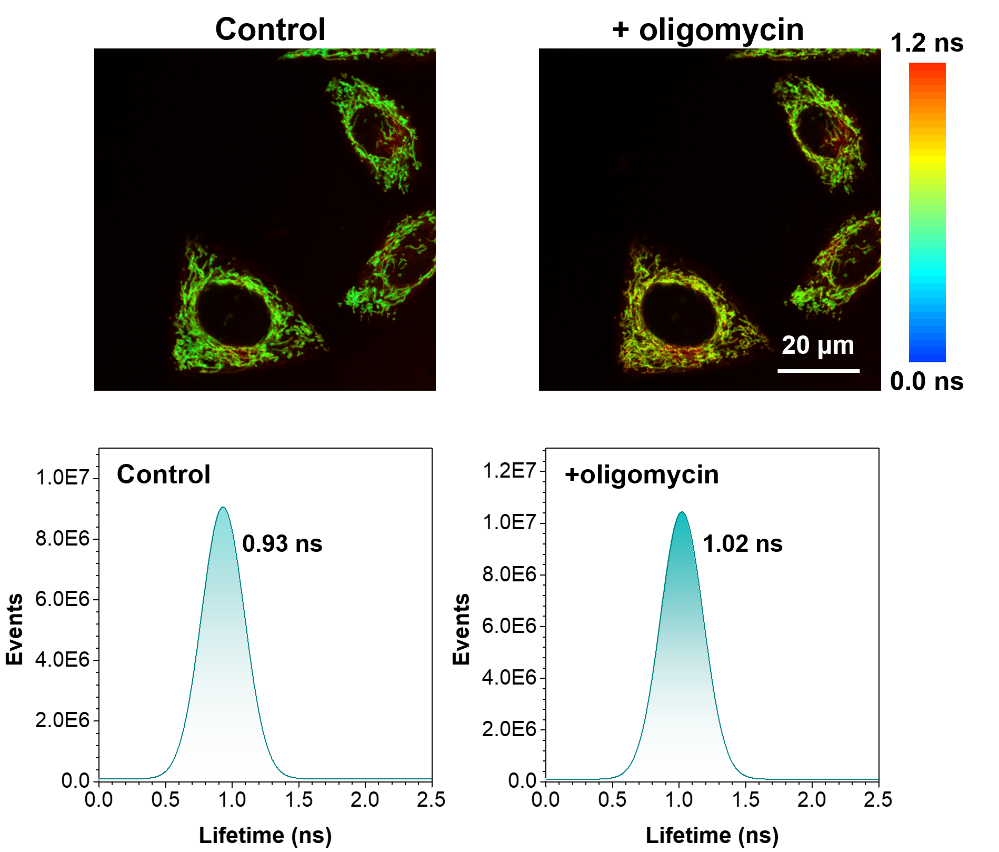


**Figure S21.** FLIM images and fluorescence lifetime histograms of HeLa cells stained with OTS-12C treatment with or without oligomycin.


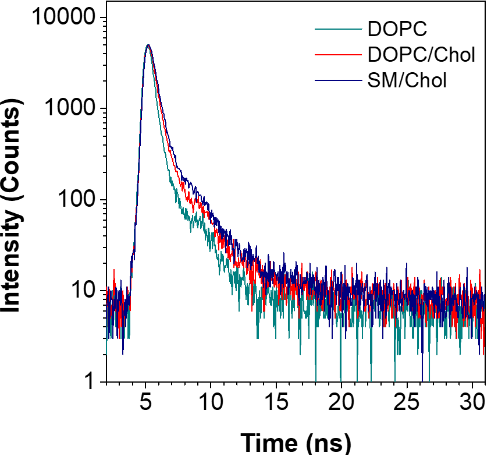


**Figure S22.** Fluorescence decay of OTS-12C in the lipid bilayer of liposomes composed of DOPC, DOPC/Chol (7/3), and SM/Chol (7/3).
